# Ubiquitination switches EphA2 vesicular traffic from a continuous safeguard to a finite signalling mode

**DOI:** 10.1038/ncomms9047

**Published:** 2015-08-21

**Authors:** Ola Sabet, Rabea Stockert, Georgia Xouri, Yannick Brüggemann, Angel Stanoev, Philippe I. H. Bastiaens

**Affiliations:** 1 Department of Systemic Cell Biology, Max Planck Institute of Molecular Physiology, Otto-Hahn-Strasse 11, 44227 Dortmund, Germany; 2 Faculty of Chemistry and Chemical Biology, TU Dortmund, Otto-Hahn-Strasse 6, 44227 Dortmund, Germany

## Abstract

Autocatalytic phosphorylation of receptor tyrosine kinases (RTKs) enables diverse, context-dependent responses to extracellular signals but comes at the price of autonomous, ligand-independent activation. Using a conformational biosensor that reports on the kinase activity of the cell guidance ephrin receptor type-A (EphA2) in living cells, we observe that autonomous EphA2 activation is suppressed by vesicular recycling and dephosphorylation by protein tyrosine phosphatases 1B (PTP1B) near the pericentriolar recycling endosome. This spatial segregation of catalytically superior PTPs from RTKs at the plasma membrane is essential to preserve ligand responsiveness. Ligand-induced clustering, on the other hand, promotes phosphorylation of a c-Cbl docking site and ubiquitination of the receptor, thereby redirecting it to the late endosome/lysosome. We show that this switch from cyclic to unidirectional receptor trafficking converts a continuous suppressive safeguard mechanism into a transient ligand-responsive signalling mode.

## Abstract (web summary)

Receptor tyrosine kinases can be auto-activated independent of ligand. Sabet *et al.* show that on ligand stimulation, ephrin receptor EphA2 switches from auto-activation suppression by vesicular recycling to ligand-dependent signalling limited by directional trafficking to late endosomes/lysosomes.

## Graphical abstract

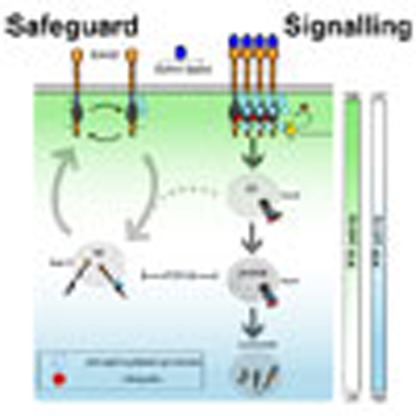

Eph receptors constitute the largest subfamily of receptor tyrosine kinases (RTKs) and together with their ephrin ligands function as principal cell guidance cues during development and disease^1^,^2^,^3^. Eph/ephrin interactions normally lead to cell–cell repulsion and sorting, yet depending on the cellular context, the same pair can elicit opposite effects^4^,^5^. Activation of Ephs follows a general RTK framework beginning with ligand binding, receptor oligomerization and subsequent trans-autophosphorylation catalysed by the kinase domain^6^. Tyrosine phosphorylation of two residues in the conserved juxtamembrane segment (JMS) and a third one in the kinase activation loop triggers conformational changes that release inhibitory interactions between the JMS and kinase domain. These regulatory tyrosines can directly enhance Eph catalytic activity by modulating the structure of the kinase domain to allow ATP and substrate to access the active site^7^,^8^,^9^,^10^.

Activation of RTKs by autophosphorylation is thus an autocatalytic system that generates an amplified response to an extracellular signal. However, the classical view of RTK activation does not take into account the conformational plasticity of the kinase domain, random receptor collisions on membranes and the fraction of enzyme already in an active state^11^, all of which are factors that can lead to spurious phosphorylation and uncontrolled receptor activity. Indeed, EphA2 expression and activity are elevated in clinical specimens of human cancer, including that of colon, breast, prostate and aggressive melanomas^12^,^13^,^14^.

Such an autocatalytic RTK system needs to be counterbalanced by the opposing activity of protein tyrosine phosphatases (PTPs)^15^. Consistent with this, elevated PTP activity in EphA3-positive leukaemia cells maintains a dephosphorylated receptor that provokes adhesion to an ephrin-A5 surface, while PTP inhibition induces receptor phosphorylation resulting in a repulsive response^16^. Eph-specific PTPs include PTP receptor type O^17^, the leukocyte common antigen related receptor tyrosine phosphatase (LAR-1)^18^ and the endoplasmic reticulum-anchored PTP1B^19^,^20^. Considering that the catalytic activity of fully active PTPs is up to three orders of magnitude higher than RTKs^21^,^22^, a gradient of PTP activity originating in the perinuclear area and declining towards the plasma membrane (PM)-proximal cytoplasm is a prerequisite for allowing RTK signal initiation^23^,^24^. However, the low PTP activity near the PM would imply that autonomous RTK activation and spurious signals are likely to occur at high surface density^25^,^26^. The density of receptors at the cell surface is dynamically controlled through a balance between endocytic uptake and vesicular recycling. Typically, ligand binding triggers receptor internalization into Rab5-positive early endosomes that mature into sorting endosomes or multivesicular bodies (MVBs). Sorting endosomes/MVBs gradually develop into late endosomes that are enriched in proteins such as Rab7. Fusion of late endosomes with lysosomes leads to receptor degradation and signal attenuation. Ligand-activated RTKs can also recycle back to the PM from peripheral endosomes or from the Rab11-positive pericentriolar recycling endosome (RE) resulting in sustained signalling^27^,^28^. This indicates that the endocytic system, positioned spatially and temporally between the PM and the lysosomes, can control RTK signal duration. Despite ample studies on membrane trafficking of ligand-activated RTKs, little is known about the role of trafficking in regulating autonomous RTK activity, an important facet considering the propensity of RTKs to self-activate. To study how vesicular membrane dynamics differentially control the activity of autonomously-versus ligand-activated EphA2, we designed a genetically encoded fluorescent biosensor for EphA2 that enables the determination of the fraction of active receptor in live cells by imaging its fluorescence lifetime. This allows for the quantification of EphA2 autocatalytic response properties, whereby we show that a ubiquitin-mediated switch in receptor trafficking converts cyclic spurious activity suppression into unidirectional ligand-mediated signal propagation.

## Results

### A genetically encoded conformational biosensor for EphA2

To investigate the dependence of EphA2 activation on its local density on cellular membranes, we engineered a genetically encoded Förster Resonance Energy Transfer (FRET) biosensor that reports its active conformation. In its non-phosphorylated form, the Eph-JMS adopts a conformation that hinders the kinase domain from adopting an ordered, active structure. Autophosphorylation of two tyrosine residues in the JMS causes a conformational change that frees the kinase domain from the inhibitory JMS and allows it to adopt an active state^7^,^8^.

**Figure 1:**
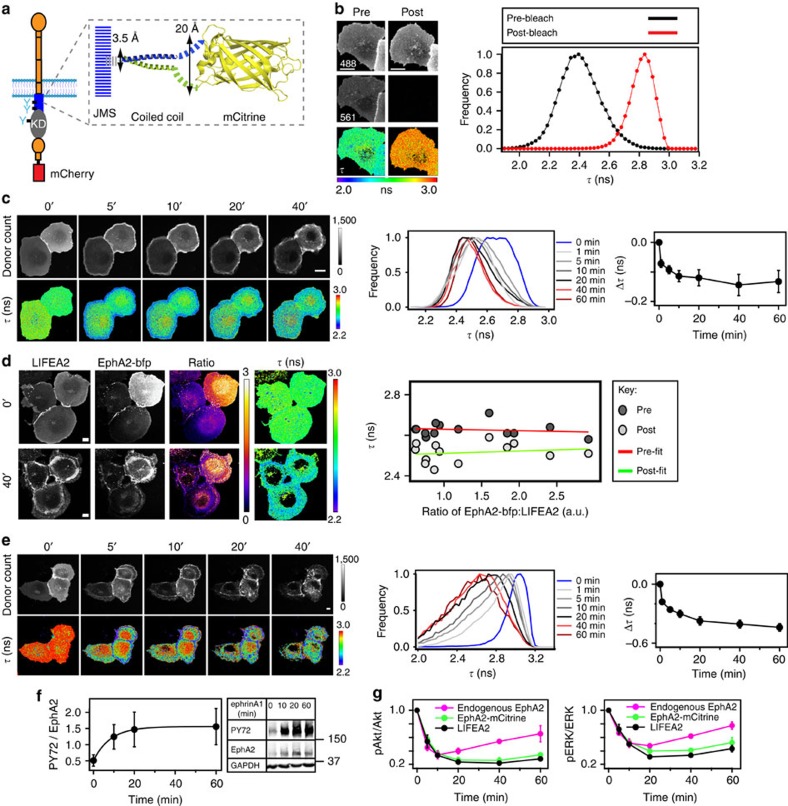
The EphA2 conformational sensor LIFEA2. (**a**) LIFEA2 consists of mCherry fused to EphA2 C-terminus and mCitrine inserted in the JMS. Inset: mCitrine JMS insertion via a coiled-coil linker that on one end matches the distance between two amino acids (3.5 Å) and on the other end matches mCitrine termini (20 Å) (PDB: 1EMA). (**b**) Fluorescence lifetime (*τ*) reports FRET in LIFEA2. First row: mCitrine fluorescence, second: mCherry fluorescence, third: *τ* (ns; color-coding below). First column: pre-mCherry photobleaching; second column: post-mCherry photobleaching. Graph: *τ*-histograms pre- and post-photobleaching. (**c**) *τ* of LIFEA2 on ephrinA1 stimulation in Cos-7 cells at the indicated time (min). Left images, upper row: mCitrine photon counts (donor count), lower row: *τ* (ns; color-coding right). Middle graph: corresponding *τ*-histograms (color-coding right). Right graph: relative drop in *τ* (Δ*τ*, ns) after LIFEA2 stimulation with pre-clustered ephrinA1-Fc (2 μg ml^−1^) (mean±s.e.m.). Data obtained form three independent experiments, *n*=7 cells. (**d**) LIFEA2 reports on a change in receptor conformation. First column: fluorescence images of LIFEA2, second: fluorescence images of EphA2–bfp, third: ratio of EphA2–bfp/LIFEA2, (arbitrary units; color-coding right), fourth: *τ* (ns; color-coding right), before (upper row) and 40 min after (lower row) addition of pre-clustered ephrinA1-Fc (2 μg ml^−1^). Graph: 2D histogram of *τ* versus EphA2–bfp/LIFEA2 ratio in single cells (*n*=13 cells) pre- and 40 min post-stimulation and the corresponding linear fits. (**e**) Phosphorylation of LIFEA2 measured by its interaction with dSH2-mCherry on ephrinA1 addition for the indicated time (min). Left images, upper row: mCitrine photon counts (donor count), lower: *τ* (ns; color-coding right) of donor-only LIFEA2 (lacks the C-terminal mCherry). Middle graph: corresponding *τ*-histograms (color-coding right). Right graph: relative drop in *τ* (Δ*τ*, ns) after LIFEA2 stimulation with pre-clustered ephrinA1-Fc (2 μg ml^−1^) (mean±s.e.m.). Data obtained form three independent experiments, *n*=8 cells. (**f**) Western blot of anti-GFP IP LIFEA2 following stimulation with pre-clustered ephrinA1-Fc (2 μg ml^−1^). Blots probed with anti-phospho-tyrosine (PY72), anti-EphA2 and anti-GAPDH antibodies. Graph: phosphorylated fraction of EphA2 (PY72/EphA2)±s.e.m. from three blots. (**g**) Downstream signalling of endogenous EphA2, EphA2-mCitrine and LIFEA2 in Cos-7 cells on ephrinA1-Fc addition for the indicated time (min). Phosphorylated fraction of Akt (pAkt/Akt) and ERK1/2 (pERK/ERK)±s.e.m. from three blots. Scale bars: 10 μm.

To monitor this conformational change by FRET, a monomeric citrine (mCitrine) was inserted with two linkers in the EphA2-JMS and a monomeric cherry (mCherry) was fused to the C-terminus of EphA2. The linkers were designed to form an antiparallel coiled-coil helix that: (1) constrains the orientation of mCitrine with respect to the insertion point, (2) minimally perturbs the JMS and (3) positions the mCitrine on ‘a stalk' outside the body of EphA2 to avoid hindrance of EphA2 interactions (Fig. 1a). Conformational changes in this Linker optimized Intramolecular-FRET sensor for EphA2 (LIFEA2) alter the FRET efficiency between the two fluorescent proteins (FP), which can be quantified via the fluorescence lifetime (*τ*) of mCitrine using fluorescence lifetime imaging microscopy (FLIM)^29^. The fluorescence lifetime of LIFEA2 expressed in Cos-7 cells exhibited a lower *τ* as compared with that of mCitrine (2.92±0.07 ns at 37 °C)^30^, suggesting the occurrence of FRET in the construct. This was confirmed by photobleaching mCherry in live Cos-7 cells, where the resultant *τ* of LIFEA2 approached that of mCitrine (Fig. 1b). Stimulation with clustered ephrinA1-Fc (2 μg ml^−1^) induced a rapid translocation of LIFEA2 to the PM accompanied by a marked reduction of its fluorescence lifetime at the cell periphery. This was followed by the internalization of LIFEA2 in its ligand-induced state to the interior of the cell (Fig. 1c). To assess whether the change in the fluorescence lifetime of LIFEA2 reports on a change in receptor conformation (intramolecular FRET) and not clustering (intermolecular FRET), the amount of LIFEA2 at the PM was diluted by co-expressing EphA2 C-terminally tagged with a blue fluorescent protein (EphA2–bfp). The expression of EphA2–bfp can be assessed by its blue fluorescent emission that is invisible in the spectral window of LIFEA2. The independence of LIFEA2 fluorescence lifetime from EphA2–bfp expression in both stimulated and resting cells showed that the drop in *τ* reports on a conformational change in LIFEA2 and not receptor clustering (Fig. 1d). This was further confirmed by two independent experiments that demonstrated that LIFEA2 clustering as measured by fluorescence anisotropy is not affecting its fluorescence lifetime (Supplementary Fig. 1a,b and Supplementary Note 1).

From the ligand-induced interaction of a LIFEA2 variant that only has the mCitrine JMS insertion with mCherry-labelled dimeric SH2 domain of pp60^src^ (dSH2-mCherry)^31^ we conclude that the drop in LIFEA2 fluorescence lifetime reflects a conformational transition to a state that has a phosphorylated JMS (Fig. 1e). This was also reflected in the phosphorylation profile of LIFEA2 that followed its conformational activation kinetics (Fig. 1f). LIFEA2 maintained its native autophosphorylation activity as well as its capacity to inhibit the PI3K/Akt and Ras/ERK signalling pathways^32^,^33^, but exhibited a more prolonged signalling response as compared with endogenous EphA2 (Fig. 1g and Supplementary Fig. 1c–e). However, the comparable ERK and Akt signalling profiles of the C-terminally tagged EphA2-mCitrine show that the insertion of mCitrine in the JMS does not alter the intrinsic activation mechanism of the receptor (Fig. 1g). Rather, as we will show later on, the more sustained response results from the higher ectopic expression of LIFEA2 that is not as efficiently cleared from the PM by the activity of the endogenous ubiquitin E3-ligase c-Cbl^34^,^35^,^36^. Taken together, the drop in fluorescence lifetime of LIFEA2 reflects the activation of the receptor in live cells by reporting the transition from a catalytically inactive conformation with high *τ* to a catalytically active state with low *τ*. In the absence of stimulus, we observed these lower fluorescence lifetimes in cells with a high expression level of LIFEA2 (compare the two cells in Fig. 1c). This was also reflected by the broad distribution of *τ* values in resting cells (Fig. 1c, blue histogram) and indicative of autonomous activation of EphA2 at high receptor expression levels.

### Autonomous activation of EphA2

**Figure 2:**
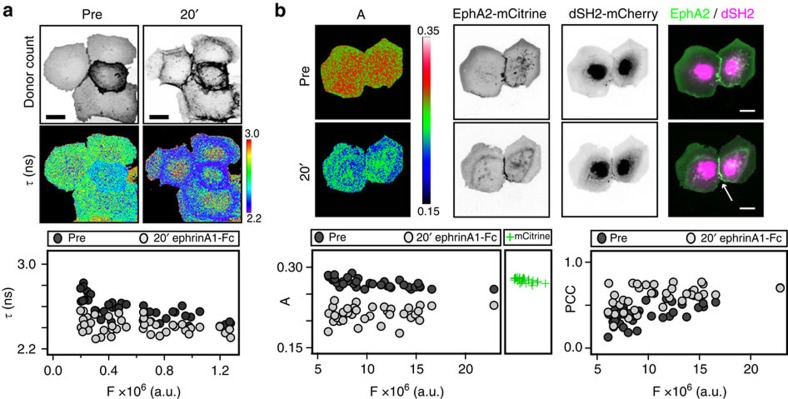
Autonomously activated EphA2 is a monomer. (**a**) Expression level dependence of LIFEA2 fluorescence lifetime (*τ*). Upper images: representative fluorescence- and *τ*-images of LIFEA2 expressed in Cos-7 cells. Upper row: mCitrine donor counts from LIFEA2, lower: *τ* (ns; color-coding right). First column: pre-stimulation; second: 20 min post-stimulation with pre-clustered ephrinA1-Fc, 2 μg ml^−1^. Lower graph: 2D histogram of *τ* versus integrated mCitrine fluorescence intensity (*F*) for individual cells (*n*=54 cells) pre- (dark grey circles) and 20 min post-stimulation (light grey circles). (**b**) Expression level dependence of EphA2-mCitrine anisotropy. Upper images: representative fluorescence- and anisotropy-images of EphA2-mCitrine expressed in Cos-7 cells. First column: anisotropy images (A) of EphA2-mCitrine (color-coding right), second: EphA2-mCitrine fluorescence, third: dSH2-mCherry fluorescence, fourth: merge of EphA2-mCitrine (green) and dSH2-mCherry (magenta) fluorescence (white arrow points at colocalization). Upper row: pre-stimulation, lower: 20 min post-stimulation with pre-clustered ephrinA1-Fc, 2 μg ml^−1^. Lower graphs: left: 2D histogram of A versus *F* for individual cells (*n*=41 cells) pre- (dark grey circles) and 20 min post-stimulation (light grey circles), right box: anisotropy of cytoplasmic mCitrine measured in multiple cells (*n*=27 cells) (+), mean±s.e.m. (0.273±0.004). Right: 2D histogram of Pearson's correlation coefficient (PCC) for colocalization versus *F* for individual cells pre- (dark grey circles) and 20 min post-stimulation (light grey circles). Scale bars: 10 μm.

To obtain information about the autonomous response properties of EphA2, the cell-to-cell variance in LIFEA2 expression was exploited by relating the conformational state of LIFEA2 (reported by *τ*) to its expression level as given by the integrated fluorescence intensity in each cell (*F*). The two-dimensional (2D) histogram of *F* versus *τ* shows that with increasing receptor expression, *τ* of unstimulated LIFEA2 approaches that of an active conformation (Fig. 2a). The abrupt change in fluorescence lifetime with LIFEA2 expression level is indicative of an autocatalytic mechanism where phosphorylation is amplified above a threshold level of tyrosine kinase activity. This EphA2 autonomous activation was corroborated by the increase in JMS phosphorylation as a function of LIFEA2 expression levels (Supplementary Fig. 2a). To estimate the variance in LIFEA2 expression level relative to endogenous EphA2, the mean of its fluorescence intensity distribution of the pixels accumulated from many cells was related to its average expression level as determined by western blot. While the mode of the asymmetric fluorescence intensity distribution showed that most cells expressed LIFEA2 at ∼2 times endogenous EphA2 levels, the variance in LIFEA2 expression ranged from ∼0.2 to ∼9 times endogenous EphA2 levels (Supplementary Fig. 2b).

Interactions between EphA2 ectodomains can form extended arrays in the presence or absence of ephrin^37^,^38^. To exclude that receptor clustering *per se* could activate EphA2 independent of an autocatalytic mechanism, we quantified the extent of EphA2-mCitrine clustering using anisotropy microscopy^39^. In the absence of ligand, highly expressed EphA2-mCitrine was phosphorylated as shown by its colocalization with dSH2-mCherry, but remained monomeric as evident from its high anisotropy that only decreased on ligand-induced clustering (Fig. 2b).

### EphA2 vesicular recycling safeguards against auto-activation

**Figure 3:**
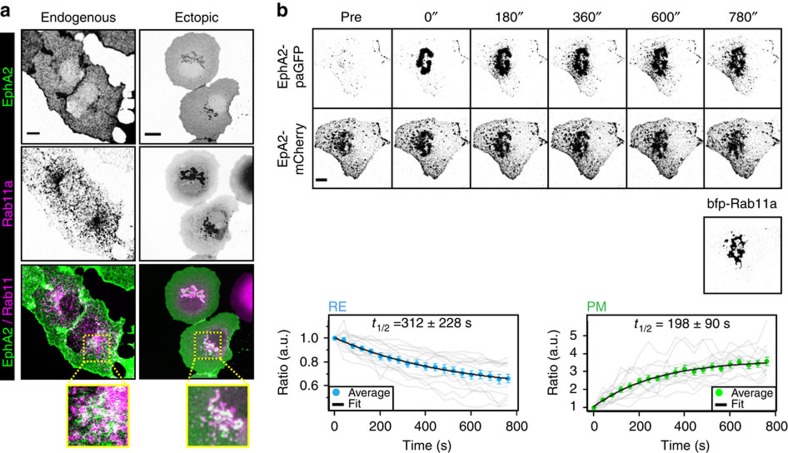
Recycling of unliganded EphA2 through the RE. (**a**) Colocalization of EphA2 and Rab11a. Left column: immunostaining of endogenous EphA2 (upper) and Rab11a (middle) in fixed Cos-7 cells, green/magenta overlay (lower). Right column: fluorescence images of ectopically expressed EphA2-mCitrine (first) and bfp–Rab11a (second) in living Cos-7 cells, green/magenta overlay (third). Fourth row represents magnified insets from respective overlay ROIs (yellow boxes). (**b**) Fluorescence redistribution after photoactivation of EphA2–paGFP in Cos-7 cells. First row: EphA2–paGFP fluorescence at the indicated times (s) after photoactivation on the RE, second: EphA2-mCherry fluorescence, third: bfp–Rab11a fluorescence. Lower graphs: ratiometrically determined decrease of EphA2–paGFP/EphA2-mCherry fluorescence at the RE (left) and gain at the PM (right). An average of 14 independent fluorescence loss and recovery curves (grey) were fitted to an exponential function to retrieve half-times (*t*_1/2_). Scale bars: 10 μm.

The signalling activity of Eph receptors is shutdown by the dephosphorylating activity of Eph-specific PTPs. The endoplasmic reticulum-anchored Eph-specific PTP1B^19^,^20^ exhibits its highest activity in the perinuclear area^23^,^40^ and can only reach the PM at specialized regions^20^,^41^. The ability of PTP1B to dephosphorylate RTKs therefore requires receptor trafficking between the PM and perinuclear membranes^23^,^40^. To assess whether unliganded EphA2 can also reach these perinuclear membranes, we first investigated if receptors recycle via the Rab11-positive pericentriolar RE^42^. Endogenous EphA2 and Rab11a exhibit partial colocalization that was increased on ectopic expression of blue fluorescent protein tagged Rab11a (bfp–Rab11a) (Fig. 3a). Fluorescence loss after photoactivation (FLAP) of EphA2 fused to photoactivatable green fluorescent protein (EphA2–paGFP)^43^ on the RE demonstrated that EphA2 exported from the RE (*k*_offRE_=2.1±1.5 × 10^−3^ s^−1^) concurrently appears on the PM with similar kinetics (*k*_onPM_=3.5±1.5 × 10^−3^ s^−1^) (Fig. 3b). Fluorescence recovery after photobleaching of EphA2-mCitrine on the RE showed that EphA2 is also trafficking from the PM back to the RE (*k*_onRE_=4.8±1.5 × 10^−3^ s^−1^) (Supplementary Fig. 3).

**Figure 4:**
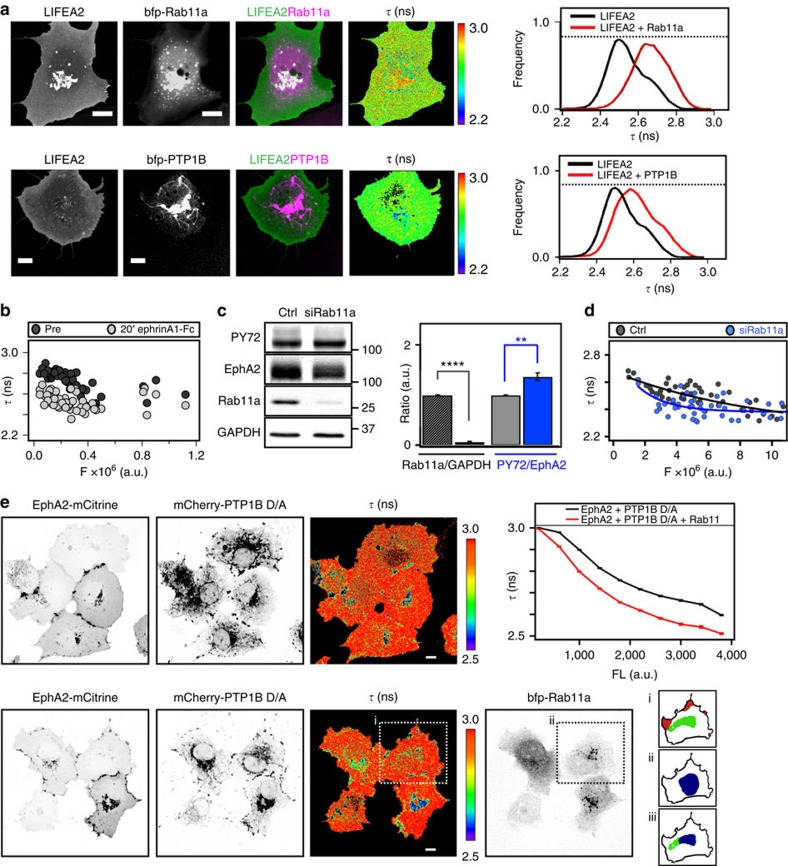
Vesicular recycling suppresses autonomous activation of EphA2. (**a**) Ectopic expression of Rab11a or PTP1B suppresses autonomous activation of LIFEA2 in Cos-7 cells. For each panel: LIFEA2 fluorescence (first column), bfp–Rab11a or bfp–PTP1B fluorescence (second), overlay of LIFEA2 (green) and bfp–Rab11a or bfp–PTP1B (magenta) (third) and LIFEA2 *τ*-maps (fourth), color-coding, right. Right graphs: *τ*-histograms from *n*=3 experiments without (*n*=7 cells) or with bfp–Rab11a (upper, *n*=8 cells) or bfp–PTP1B (lower, *n*=7 cells) expression. (**b**) Expression level dependence of LIFEA2 fluorescence lifetime (*τ*) on ectopic expression of bfp–Rab11a. 2D histogram of *τ* versus integrated fluorescence intensity (*F*) for individual cells (*n*=54 cells) pre- (dark grey circles) and 20 min post-stimulation (light grey circles). (**c**) EphA2 phosphorylation is increased by Rab11a knockdown Left: western blot of Cos-7 lysates 72 h post-transfection with non-targeting siRNA (Ctrl, first column) or Rab11a siRNA (siRab11a, second column). Blots were probed for phospho-tyrosines (PY72, first row), EphA2 (second row), Rab11a (third row) and GAPDH (fourth row). Graph: ratio of Rab11a to GAPDH (*n*=5), and PY72 to total EphA2 (*n*=4) [mean±s.e.m., **P<0.01; ****P<0.0001; unpaired *t*-test]. (**d**) Expression level dependence of LIFEA2 fluorescence lifetime (*τ*) on Rab11a knockdown in Cos-7 cells. 2D histogram of LIFEA2 *τ* versus its integrated fluorescence intensity (*F*) for individual cells (*n*=49 cells) with non-targeting siRNA (Ctrl) or Rab11a siRNA (siRab11a). The two distributions fitted with an exponential function (black and blue lines) showed a statistical difference with *P* value=0.034 [*P<0.05; Kolmogorov–Smirnov test]. (**e**) Interaction of EphA2-mCitrine with substrate-trapping mutant PTP1B D/A-mCherry detected by FLIM–FRET in the absence (upper panel) or presence (lower panel) of bfp–Rab11a. First column: fluorescence images of EphA2-mCitrine, second: fluorescence images of mCherry–PTP1B D/A, third: *τ* (ns; color-coding right) of EphA2-mCitrine, fourth: fluorescence image of bfp–Rab11a. Graph: average fluorescence lifetime of EphA2-mCitrine (*τ*)±s.e.m. versus its fluorescence intensity (FL) in absence (black, *n*=55 cells) or presence (red, *n*=50 cells) of bfp–Rab11a expression. The percentage of EphA2-mCitrine/PTP1B-D/A-mCherry interactions in the vicinity of Rab11a-positive endosomes (iii) was retrieved from the overlap between non-cell contact areas with low *τ* values (i, green) and high intensity areas in bfp–Rab11a images (ii, blue). Scale bars, 10 μm.

To assess whether recycling EphA2 through the perinuclear, active pool of PTP1B suppresses autonomous phosphorylation, bfp–Rab11a or bfp–PTP1B were ectopically expressed and the conformational state of LIFEA2 was measured. In both cases, the *τ*-distribution of LIFEA2 was shifted towards that of an inactive receptor (Fig. 4a). This was corroborated by a decrease in the basal phosphorylation of endogenous EphA2 on expression of bfp–PTP1B or bfp–Rab11a (Supplementary Fig. 4a). Bfp–Rab11a expression also increased the amount of LIFEA2 at which autonomous activation occurred (Fig. 4b), and lowered the phosphorylation of EphA2-mCitrine (Supplementary Fig. 4b). Knockdown of Rab11a had the opposite effect of increasing phosphorylation of endogenous EphA2 (Fig. 4c) and lowering the expression level at which LIFEA2 was autonomously activated (Fig. 4d).

To determine where autonomously activated EphA2 is dephosphorylated by PTP1B, we examined EphA2–PTP1B interactions with the substrate-trapping mutant of PTP1B (PTP1B-D181A). The catalytically impaired PTP substrate-trapping mutants retain substrate binding^44^ and can be used to identify PTP-substrate complexes in the cell^24^. By detecting FRET between EphA2-mCitrine and mCherry-tagged PTP1B-D181A (mCherry–PTP1B D/A), the sites of EphA2-mCitrine/PTP1B-D181A-mCherry interaction (low *τ*) were observed at stabilized cell–cell-contact points and in the perinuclear region (Fig. 4e). The mean spatial coincidence of EphA2/PTP1B-D181A interacting in the perinuclear region and bfp–Rab11a endosomes (81±17%) shows that PTP1B dephosphorylates EphA2 in the vicinity of the RE (Fig. 4e, Supplementary methods and Supplementary Fig. 8). Ectopic expression of bfp–Rab11a increased the amount of EphA2 that interacted with PTP1B D/A (lowered *τ*), possibly due to Rab11a-mediated biogenesis of the RE that increased the amount of EphA2 in the perinuclear area (Fig. 4e, compare red and black traces in graph).

### Ubiquitination switches trafficking of ligand-bound EphA2

**Figure 5:**
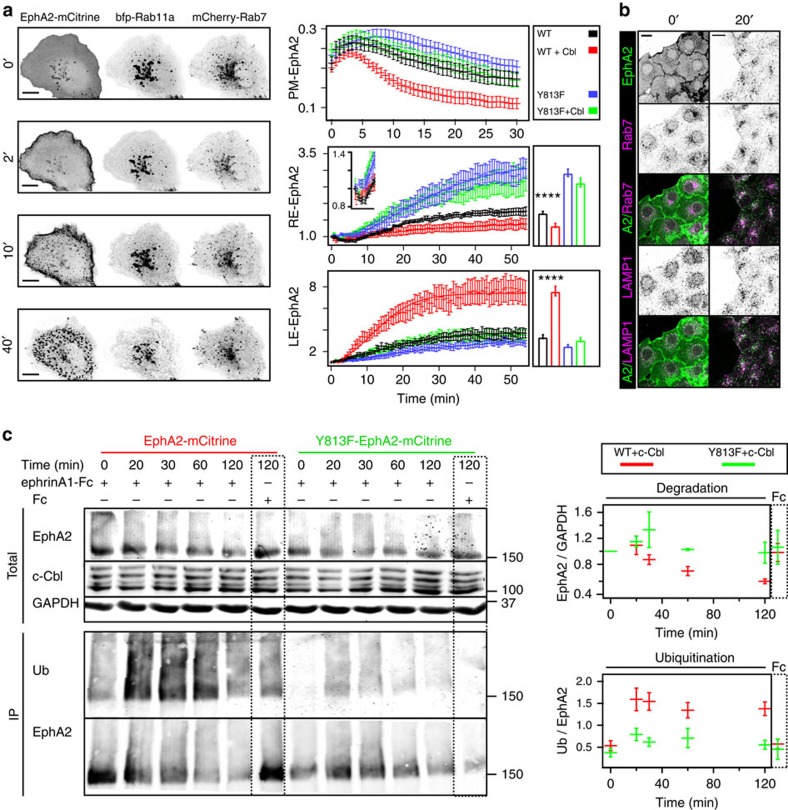
c-Cbl-mediated ubiquitination redirects ephrin-clustered EphA2 toward the LE. (**a**) Time-lapse of EphA2 and Rab11a/Rab7 colocalization in Cos-7 cells on ephrinA1 stimulation. Left panels: fluorescence images of EphA2-mCitrine (left column), bfp–Rab11a (middle), mCherry-Rab7 (right), at indicated time in min. Right graphs: ratiometric analysis of EphA2-mCitrine (WT) and Y813F-EphA2-mCitrine (Y813F) localization on different membrane compartments, with and without ectopic expression of c-Cbl (Cbl). Upper: PM (PM-EphA2), middle: Rab11a-positive RE (RE-EphA2, inset: blow-up of the first 10 min), lower: Rab7-positive late endosome (LE-EphA2). Bar graphs: average amplitude between 48–53 min±s.e.m. (*n*=12–18 cells from at least three independent experiments). Upper right box: color-coding. (**b**) Immunostaining of endogenous EphA2, Rab7 and LAMP1 in Cos-7 cells before (left column) and 20 min post-stimulation of Cos-7 cells with pre-clustered ephrinA1-Fc, 2 μg ml^−1^ (right column). EphA2 (first row), Rab7 (second row), EphA2 (green) and Rab7 (magenta) overlay (third row), LAMP1 (fourth row), EphA2 (green) and LAMP1 (magenta) overlay (fifth row). Scale bars: 10 μm. (**c**) c-Cbl induces EphA2 ubiquitination and degradation. Left: western blot of Cos-7 total cell lysate (Total, upper) and anti-GFP IP EphA2-mCitrine or Y813F-EphA2-mCitrine (IP, lower) showing time course of stimulation with pre-clustered ephrinA1-Fc (2 μg ml^−1^). Pre-clustered Fc fragment (Fc, 2 μg ml^−1^) was used as control (dashed box). c-Cbl-bfp and HA-Ubiquitin were co-expressed. Total lysates were probed with anti-EphA2, anti-c-Cbl and anti-GAPDH antibodies. IP was probed with anti-HA for ubiquitin (Ub) and anti-EphA2 antibodies. Right graphs: upper, ratiometric quantification of EphA2/GAPDH from blots of EphA2 (red) or Y813F-EphA2 (green) (mean±s.e.m. normalized to zero time point, *n*=3 blots). Lower: ratiometric quantification of Ub (ubiquitin)/EphA2 from 3 blots of anti-GFP IP EphA2 (red) or Y813F-EphA2 (green), right boxes: control Fc fragment (120 min).

We next addressed if and how this cyclic trafficking of monomeric EphA2 is affected by ligand stimulation. EphrinA1 induced a transient translocation of EphA2-mCitrine to the PM (Fig. 5a) followed by its clearance and subsequent appearance in Rab5-positive early endosomes^45^ (Supplementary Movie 1). Immunofluorescence of endogenous EphA2 and Rab5 confirmed this extensive colocalization after ephrinA1-Fc stimulation (Supplementary Fig. 5a). Quantification of the kinetics of ligand-induced EphA2-mCitrine translocation to the PM showed an initial enrichment (peak ∼5 min), paralleled by a loss of EphA2 at the RE (Fig. 5a). This transient PM-trapping of EphA2 likely originates from a shift in its steady-state distribution caused by a continuation of its vesicular trafficking from the RE and delayed endocytosis from the PM. To compare this with the trafficking of autonomously activated EphA2, PTPs were inhibited by the thiol-reactive pervanadate^46^. Contrary to ligand-activated EphA2, which slowly colocalized with the Rab7-positive late endosomes^47^ and the lysosomal-associated membrane protein-1 (LAMP-1)^48^ (Fig. 5b), autonomously activated (monomeric) receptors continued to traffic via the RE (Supplementary Fig. 5b,c). This shows that ligand-induced clustering of EphA2 causes a switch in its vesicular trafficking from recycling to unidirectional endocytosis to the LE.

**Figure 6:**
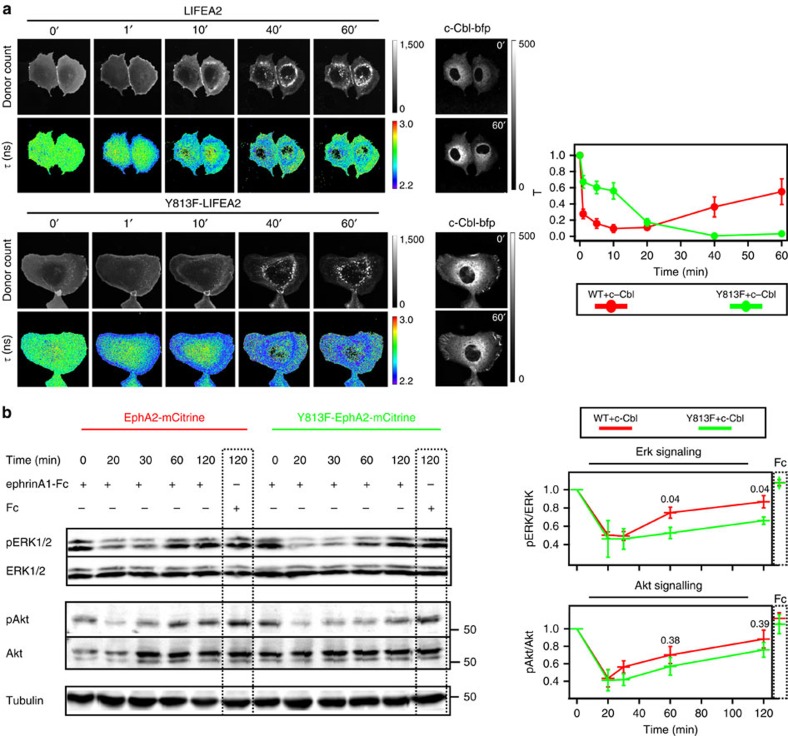
c-Cbl-mediated ubiquitination causes a transient EphA2 response. (**a**) *τ*-maps of LIFEA2 and Y813F-LIFEA2 in Cos-7 cells on ephrinA1 stimulation (time; min). Upper panel: LIFEA2, lower: Y813F-LIFEA2. For each panel, upper row: mCitrine photon counts, lower: *τ* (ns; color-coding). Right: fluorescence images of c-Cbl-bfp. Right graph: normalized fluorescence lifetime; T=(*τ*–*τ*_2_/*τ*_1_–*τ*_2_)±s.e.m. of LIFEA2 (red) or Y813F-LIFEA2 (green), where *τ*_1_ is the initial and *τ*_2_ is the smallest fluorescence lifetime value. Data obtained from three independent experiments, *n*=7 cells. Scale bars: 10 μm. (**b**) Sustained downstream signalling of Y813F-EphA2-mCitrine (green) as compared with EphA2-mCitrine (red) in Cos-7 cells co-expressing c-Cbl-bfp on ephrinA1-Fc (2 μg ml^−1^) addition for the indicated time (min). In both cases, blots of total Cos-7 lysates were probed for phospho-Erk1/2 (pErk 1/2), total Erk1/2 (Erk1/2), phospho-Akt (S473) (pAkt) and total Akt (Akt). Right graphs: normalized ratio of phospho/total Erk1/2 (upper), or phospho/total Akt (lower)±s.e.m. from four independent blots. Color-coding above graphs (*P* values depicted above the compared time points; unpaired *t*-test). Pre-clustered Fc fragment (Fc, 2 μg ml^−1^) was used as control (dashed box).

Because ephrin stimulation induces Eph ubiquitination by the E3-ligase c-Cbl^34^,^35^,^36^, we investigated whether this regulates the differential trafficking of ligand-clustered receptors as compared with autonomously activated EphA2. Quantification of EphA2-mCitrine fluorescence on ectopic expression of c-Cbl revealed faster PM clearance and more pronounced trafficking towards the LE in response to ephrin stimulation (Fig. 5a). The SH2 domain of Cbl binds to specific phosphorylated tyrosine residues on RTKs in the consensus Cbl-docking sequence (YXXXP)^49^, which in EphA2 would correspond to the yet uncharacterized tyrosine 813 (refs 34, 50, 51). Mutation of this tyrosine to phenylalanine (Y813F) strongly reduced receptor ubiquitination and slowed down its internalization. On ligand addition, Y813F-EphA2-mCitrine continued to recycle via the RE instead of being redirected to the LE, thereby preventing its degradation (Fig. 5a,c). To investigate the impact of this ubiquitination switch on EphA2 activity, the conformational response of a LIFEA2 mutant harbouring the Y813F mutation was analysed. In the absence of ligand, ectopically expressed Y813F-LIFEA2 and bfp–Rab11a exhibited clear colocalization, which shows that it recycles similarly to Y813F-EphA2-mCitrine (Fig. 5a, Supplementary Fig. 6). On ligand addition, Y813F-LIFEA2 continued to recycle even in the presence of ectopically expressed c-Cbl and this continued receptor recycling resulted in a persistent activation (Fig. 6a). In addition, we observed a more sustained inactivation of ERK downstream of Y813F-EphA2-mCitrine as compared with EphA2-mCitrine, and to a lesser extent for Akt (Fig. 6b). This demonstrates the essential role of EphA2 ubiquitination in redirecting vesicular trafficking to the LE to impose a finite signalling response to ephrin.

## Discussion

**Figure 7:**
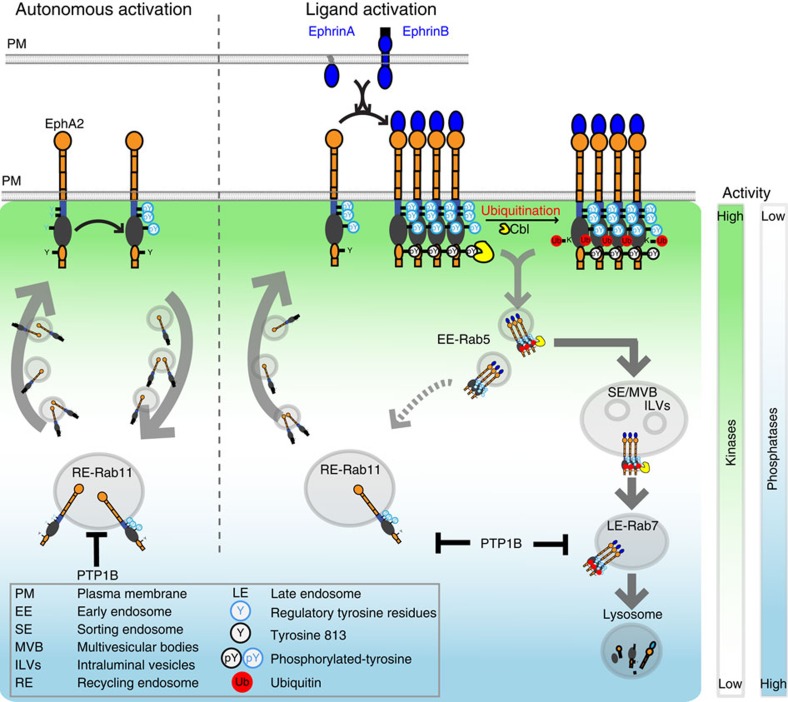
Differential trafficking of autonomously activated EphA2 versus ligand-activated EphA2. Cyclic trafficking of EphA2 via the perinuclear pool of active PTPs acts as a safeguard mechanism against its autonomous activation (left). Ligand-induced clustering and docking of c-Cbl mediates receptor ubiquitination and unidirectional trafficking through the sorting endosomes/MVBs towards the late endosomes/lysosomes, thereby ensuring a transient response to growth factors (right). Lower left box: abbreviations and symbols.

Both pro- and anti-oncogenic properties have been attributed to EphA2, with a connection between receptor amount, ligand-independent activity and malignancy^33^,^52^, yet it remained unclear how the cell suppresses autonomous receptor activation while maintaining the capacity to respond to ligand stimulation. Here we show that coupling of EphA2 self-association state, ubiquitination and vesicular dynamics allows for the coexistence of a safeguard mechanism against autonomous EphA2 activation while maintaining the capacity to transiently respond to extracellular signals (Fig. 7).

We investigated the molecular determinants that trigger the endocytic machinery to deal with autonomously activated EphA2 in a different manner than when it is ligand bound. High expression level of EphA2 or inactivation of PTPs triggered receptor phosphorylation with little internalization into early endosomes (Supplementary Fig. 5a and Supplementary Movie 3). This implies that autonomously activated receptors cannot engage the Rab5a machinery in contrary to ligand-activated receptors^53^,^54^. Autonomously activated EphA2 remained unclustered, which points to the importance of receptor clustering in differentiating between autonomously and ligand-activated EphA2 (Fig. 2b and Supplementary Fig. 5b). On the other hand, both wild-type (WT) EphA2 and its ubiquitination-deficient mutant (Y813F-EphA2) were clustered on the cell periphery on ephrin addition, thereby showing that ubiquitination has a dominant role in mediating Eph trafficking towards LE/lysosomes.

The question is at which point in the endocytic pathway the switch in EphA2 trafficking occurs. Both WT and Y813F-EphA2 were internalized into Rab5a-positive early endosomes after ligand stimulation (Supplementary Movie 1 and 2). However, since only ligand-activated Y813F-EphA2 kept recycling via the RE (Fig. 5), the ubiquitin-mediated switch in receptor trafficking must occur after entry of receptors into the early endosomes. In support of this, fusion of ubiquitin to the normally recycled transferrin receptor led to its differential sorting in early endosomes so that it is redirected to the degradation pathway^55^.

It also follows that the Cbl-docking sequence can only be efficiently phosphorylated when the receptor is clustered by ligand. Similarly, the Cbl-binding tyrosine of EGFR is not efficiently phosphorylated on autonomous receptor activation by oxidative stress^56^. Receptor clustering might affect the accessibility of Y813 to the kinase domain of EphA2, enhance the interaction of EphA2 with other tyrosine kinases like Src^35^,^51^ or hinder its accessibility to PTPs. Regardless of the mechanism underlying phosphorylation of Y813 in EphA2 clusters, Cbl-binding is a limiting step for the internalization of receptors, as seen for EGFR and Met receptors^57^,^58^. The fact that Cbl expression increased PM clearance of WT receptor and modified its signalling response from a prolonged to a transient one shows that its stoichiometric interaction with EphA2 is important for ubiquitination (Figs 5a and 6b).

As clustered receptors await Cbl-mediated ubiquitination, continuous trafficking of EphA2 from the RE to the PM results in a net translocation and concentration at the PM with a parallel depletion from the RE (Fig. 5a). The resulting change in the balance between internalized and recycled receptors causes an apparent trap of ligand-bound EphA2 on the PM. This increases the chances of Eph–Eph interactions^38^, which results in trapping further receptors^59^ and a shift in the RTK/PTP balance in favour of kinase activity at the PM. Autocatalytic RTK activity that is coupled to inhibition of PTPs, typically by reactive oxygen species, can result in an amplification of the initial ligand-activated receptor population^15^,^60^. Such a bistable RTK/PTP activation switch can be irreversible^61^, which implies that receptors have to be shutdown by trafficking them to a perinuclear area where PTP activity is not coupled to RTK activity.

We have previously shown that the activity of PTP1B is highest on perinuclear membranes^23^. Work by us and others has additionally shown that endocytosis of RTKs is necessary for their interaction with PTP1B^24^,^62^. This is essential, because a high activity of PTP1B near the PM would completely suppress EphA2 phosphorylation and thereby signalling. We have recently reported a seemingly contradictory result in which we showed that PTP1B can dephosphorylate EphA2 on the PM at points of cell–cell contact^20^. However, ligand-induced EphA2 signalling typically results in cell retraction. To maintain a preformed cell–cell contact, EphA2 phosphorylation must be actively suppressed, which is achieved by remodelling the endoplasmic reticulum to allow PTP1B to reach these stabilized cell-contact points^20^. Receptor recycling thus allows an exploring cell to suppress spurious EphA2 activation while the ubiquitin-mediated trafficking switch maintains its capacity to undergo repulsion on encountering another cell.

Receptor recycling between the PM and RE takes place on the order of 5 min, which is sufficient to suppress the slow autonomous phosphorylation of EphA2 at the PM. Why then is receptor recycling not sufficient to achieve signal termination of ligand-activated receptors? Ephrin stimulation generates higher-order clusters^63^ that mediate rapid Eph phosphorylation that cannot be countered by the relatively slow recycling on the minute timescale. Ephrin-Eph clusters have a lifetime of up to 100 min as determined by *in vitro* experiments with dimeric ephrins^64^. Trafficking of ephrinA1-EphA2 to the perinuclear area will dephosphorylate the receptor complex. However, on continued vesicular recycling it will become reactivated by rapid autophosphorylation due to the low phosphatase activity at the PM. Therefore, vesicular recycling and dephosphorylation in the perinuclear area would give rise to a sustained EphA2 response as observed for the Y813F-LIFEA2 mutant that lacks the c-Cbl docking site (Fig. 6a). It is through the unidirectional traffic of ligand-activated EphA2 toward the LE that signalling can be efficiently terminated, by restricting receptors to an area with high PTP activity until lysosomal degradation clears the system.

The key regulatory switch in vesicular trafficking of EphA2 from cyclic to unidirectional trafficking is likely shared by other RTKs, since their autocatalytic activation needs to be kept in check by PTPs^15^. This fundamental mechanism for cellular regulation of RTK activity serves the duality of signal propagation versus suppression, allowing safeguarding against autonomous activation while maintaining the capacity to elicit a finite response to ligand.

## Methods

### Molecular biology

All C-terminally tagged EphA2 constructs were generated by inserting human, full-length EphA2 (gift from T. Pawson) between AgeI and KpnI restriction sites of: mCitrine-N1, mCherry-N1 (Clontech); Tagbfp-N1 (Evrogen) or paGFP-N1 (gift from J. Lippincott-Schwartz, NIH). All FP used in this study carried the A206K mutation that renders them monomeric. EphA2-mCherry-N1 served as starting plasmid for insertion of mCitrine in the EphA2-JMS. Insertion was optimized within a stretch of 10 amino acids starting from D573, with the optimal insertion point being after S577. Several types of linkers were cloned before and after the sequence of mCitrine to further optimize its insertion into the JMS. Linker sequences were obtained from http://www.ibi.vu.nl/programs/linkerdbwww/, with the final linker sequence being LAAAYSSILSSNLSSDS. This was used to create the final chimera: N-EphA2-LAAAYSSILSSNLSSDS-mCitrine-SDSSLNSSLISSYAAAL-EphA2-C. FP fusions to Rab7, Rab11 and Rab5 were generated through restriction ligation of Rab7 and Rab5 (gift from Y. Wu, CGC Dortmund) and Rab11a (Addgene) cDNA inserts into the appropriate FP encoding vectors (mCherry-C1, Tagbfp-C1 and mCitrine-C1). All constructs were sequence verified and tested for correct expression. Fluorophore fusions of HA-c-Cbl were obtained by excision replacement of Citrine by mCherry or bfp from HA-c-Cbl-Citrine (gift from I. Dikic, iBC II, Frankfurt am Main). Mutants of EphA2 were generated by site-directed mutagenesis of the appropriate EphA2 and/or LIFEA2 constructs using the Quickchange Site-Directed-Mutagenesis kit (Stratagene). HA-Ubiquitin (gift from I. Dikic, iBC II, Frankfurt am Main) and FP fusions to PTP1B and PTP1B D/A were generated through restriction ligation of PTP1B and PTP1B-D181A (gift from B. Neel, UHN Toronto) inserts into the appropriate FP encoding vectors (Tagbfp-N1 and mCherry-N1).

### Antibodies and reagents

Primary antibodies: rabbit anti-phospho-EphA2/3/4 Y588/Y596 (pJM) (ab62256, Abcam, Cambridge, UK, 1:500); mouse anti-phospho-tyrosine (PY72) (P172.1, *in vivo* Biotech Services GMBH, 1:720), mouse anti-HA (MMS-101P, Covance, 1:1,000); rabbit anti-EphA2 (sc-924, Santa Cruz Biotechnologies, Santa Cruz, CA, 1:200); goat anti-EphA2 (AF-3035, R and D Systems, Minneapolis, MN, 1:500); mouse anti-EphA2 (IF7) (kind gift from M. Lackmann, 1:200); rabbit anti-c-Cbl (sc-170, Santa Cruz Biotechnologies, 1:1,000); mouse monoclonal anti-α-Tubulin (Sigma Aldrich, St. Louis, MO, 1:4,000); mouse monoclonal anti-GAPDH (CB-1001, Calbiochem, Merck Biosciences, Darmstadt, Germany, 1:3,000); living colours rabbit anti-GFP (632593, Clontech, Mountain View, CA, used for immunoprecipitation, 1:200); living colours mouse anti-GFP (632681, Clontech, Mountain View, CA, used in western blots, 1:1,000); rabbit anti-tRFP (AB234, Evrogen, Moscow, 1:2,000) and mouse anti-human IgG Fc fragment specific for clustering of Fc-fusion proteins (411455, Calbiochem), rabbit anti-Rab7 (9367, Cell Signaling Technology, Danvers, MA, 1:200), rabbit anti-Rab11a (2413, Cell Signaling Technology, used in immunofluorescence, 1:100), rabbit anti-Rab11a (ab65200, Abcam, used in western blots, 1:300), mouse anti-Rab11 (610656, BD Biosciences, used in immunofluorescence, 1:100), mouse anti-LAMP1 (ab25630, Abcam, 1:200), mouse anti-Rab5 (610281, BD Biosciences, 1:200), rabbit anti-phospho ERK-1/2 Thr/Tyr 202/204 (9101, Cell Signaling Technology, Danvers, MA, 1:500), mouse anti-ERK1/2 (Ab366991, Abcam, 1:1,000); rabbit anti phosphor-Akt Ser473 (9271, Cell Signaling Technology, Danvers, MA, 1:500); mouse anti-Akt (pan) (2920, Cell Signaling Technology, Danvers, MA, 1:1,000). Secondary antibodies for immunofluorescence: Alexa Fluor 647 goat anti-rabbit IgG, Alexa Fluor 647 donkey anti-mouse, Alexa Fluor 488 donkey anti-rabbit IgG, Alexa Fluor 555 donkey anti-goat (Life Technologies, 1:200). Secondary antibodies for western blots: IRDye 680/800 donkey anti-mouse IgG, IRDye 800 donkey anti-goat IgG, IRDye 680/800 donkey anti-rabbit IgG, (LI-COR Biosciences, 1:10,000). Reagents: Human IgG Fc fragment (110-HG) and mouse ephrinA1-Fc (602-A1) fusion protein (R and D Systems) were used for stimulations. For pre-clustering, Fc fragment and ephrinA1-Fc fusion proteins were incubated with mouse anti-human Fc (411455, Calbiochem) at a ratio of 5:1 for at least 30 min at room temperature (RT). The tyrosine kinase inhibitor Dasatinib (9052) was from Cell Signaling Technology. Pervanadate was freshly prepared by adding sodium orthovanadate (S6508, Sigma) to H_2_O_2_ 30% according to ref. 65.

### Cell culture and RNA interference

Cos-7 (ECACC No.: 87021302) and MDA-MB-231 (ATCC No.: HTB-26) cells were grown in Dulbecco's Modified Eagle's Medium supplemented with 10% foetal bovine serum, 2 mM L-glutamine and 1% non-essential amino acids and maintained at 37 **°**C in 5% CO_2_. MDA-MB-231 cells are an aggressive human breast cancer cell line in which EphA2 is overexpressed^12^. Transfection was done using FUGENE6 (Roche Diagnostics, Mannheim, Germany) for imaging experiments or Lipofectamine 2000 (Life Technologies) for western blots according to manufacturer's protocol. About 4–12 h before an experiment, cells were starved in growth medium containing dialyzed 0.5 % foetal bovine serum. For live cell microscopy, cells were cultured on 35 mm glass bottom dishes (MatTek, Ashland, USA) or four-well chambered glass slides (Lab-tek, Nunc). Dasatinib (final concentration: 200 nM) was diluted in dimethylsulphoxide and directly added in Dulbecco's Modified Eagle's Medium without phenol red (imaging media, P04-01163, PAN) to make final concentration of dimethylsulphoxide: 1% V/V. Transfection of Rab11a siRNA was achieved using siRNA transfection reagent (sc-29528, Santa Cruz) in special transfection medium (sc-36868, Santa Cruz). Both Rab11a siRNA: sc-36340-SH (Santa Cruz Biotechnology) or scrambled non-targeting control siRNA: sc-37007 (Santa Cruz Biotechnology) was used at a final concentration of 50 nM for 72 h before validation of knockdown. LIFEA2 was transfected 48 h post siRNA transfection and imaged 24 h later.

### Immunoprecipitation and western blotting

Cells were either lysed in ice-cold RIPA buffer (50 mM Tris pH 7.5, 150 mM NaCl, 1 mM ethylene glycol tetraacetic acid , 1 mM EDTA, 1% IGEPAL, 0.25% Na deoxycholate, 2.5 mM Na pyrophosphate, 1 mM β-glycerophosphate, 0.1 mM phenylmethyl sulphonyl fluoride) or ready made Cell Signaling buffer (9803, for siRNA experiments) supplemented with Complete Mini EDTA-free protease inhibitor (Roche Applied Science) and 1,000 μl phosphatase inhibitor cocktail 2 and 3 (P5726 and P0044, Sigma Aldrich). For ubiquitination immunoprecipitated (IP), cell lysis was performed in modified RIPA (50 mM Tris–HCl, 150 mM NaCl, 1 mM ethylene glycol tetraacetic acid, 1 mM EDTA, 1% Triton X-100, 1% sodium deoxycholate, 1% sodium dodecyl sulfate (SDS)), followed by dilution to a final SDS concentration of 0.2% (ref. 66). Following lysis, samples were subjected to sonication for 12 sec and then cleared by centrifugation for 15 min, 13,000 r.p.m. at 4 °C. For IP, equal amounts of protein lysates were incubated with anti-GFP or anti-Eph antibodies overnight at 4 °C followed by incubation for 2 h with Protein-G Sepharose (PG-Seph) beads. For western blots, PVDF membranes were incubated overnight with the respective primary antibody, followed by incubation with the species-specific secondary LI-COR antibody, and proteins were detected with Odyssey Infrared Imaging System (LI-COR Biosciences). For re-blotting, membranes were stripped for 15 min with stripping buffer (LI-COR Biosciences) at RT, blocked again with LI-COR blocking buffer (30 min) and re-probed with primary antibody.

### *In vitro* kinase assay

*In vitro* auto- and trans-phosphorylation assays were preformed as described earlier^9^. Cos-7 cells were transfected with cDNA encoding the EphA2 construct in question (endogenous EphA2 was precipitated from MDA-MB-231). Cells were lysed 18 h later and EphA2 was IP. Following IP, beads were washed three times with HNTG buffer (20 mM HEPES (pH 7.5), 150 mM NaCl, 0.1% Triton X-100, 10% glycerol, 100 μM sodium vanadate). Samples were dephosphorylated using calf intestinal phosphatase (#M0290, 0.5 U μg^−1^ protein) for 60 min at RT and then washed twice with kinase reaction buffer KRB (25 mM HEPES (pH 7.5), 2.5 mM MgCl_2_, 4 mM MnCl_2_, 100 μM sodium vanadate). Beads were resuspended in KRB containing 5 μg of acid denatured enolase (Sigma #E0379) at RT for the indicated time, before stopping the reaction by adding SDS/PAGE sample buffer and heating to 100 °C. Autophosphorylation of Eph or transphosphorylation of enolase was detected using PY72.

### Immunofluorescence

Cells were fixed with 4% paraformaldehyde in PBS (pH 7.4) for 10 min at RT. This was followed by permeabilization for 5 min with 0.1 % Triton X-100 in PBS at RT. Background staining was blocked by incubation with PBS, 2% normal serum for 30 min at RT. Primary and secondary antibodies (diluted in blocking buffer) were applied for 1 h and 30 min, respectively. Fixed cells were imaged in PBS at 25 **°**C.

### Confocal microscopy

Confocal images were recorded using an Olympus Fluoview FV1000 confocal microscope (Olympus Life Science Europa, Hamburg, Germany) equipped with a temperature controlled CO_2_ incubation chamber at 37 °C (EMBL, Heidelberg, Germany) and a × 60/1.35 NA Oil UPLSApo objective (Olympus). Fluorescent fusion proteins with bfp, mCitrine and mCherry or Alexa633-labelled secondary antibodies were excited using the 405 nm Diode-UV laser (FV5-LD05, Hatagaya), 488 nm Argon laser (GLG 3135, Showa Optronics), 561 nm DPSS laser (85-YCA-020-230, Melles Griot) and 633 nm HeNe laser (05LHP-991, Melles Griot), respectively. Detection of fluorescence emission was restricted with an Acousto-Optical Beam Splitter as follows: bfp (425–478 nm), mCitrine (498–551 nm), mCherry (575–675 nm). In all cases scanning was performed in frame-by-frame sequential mode with 2 × frame averaging. The pinhole was set between 1.5 and 2.5 Airy units.

### Ratiometric colocalization analysis

Time-lapse movies of Cos-7 cells transfected with EphA2-mCitrine and the different Rab fluorophore fusion proteins were obtained through confocal microscopy. Images were bleach-corrected and punctae representing Rab7- or Rab11-positive vesicles were segmented using ImageJ64 V1.44 (http://rsbweb.nih.gov/ij/). Masks of the RE were generated from bfp–Rab11a images, masks for the LE were generated from either bfp- or mCherry-Rab7 images and masks of the PM were generated from EphA2-mCitrine images. Quantification of EphA2 PM translocation was done by measuring the integrated EphA2-mCirine intensity on the cell periphery (ImageJ, erode function: 5 pixels) and normalized to the total EphA2-mCitrine fluorescence. To determine the endosomal fraction of EphA2, the cytoplasmic EphA2 background was estimated by applying the ImageJ ‘rolling ball algorithm' to perform background subtraction. The different EphA2 endosomal signals were generated by multiplying the mCitrine-channel fluorescence intensity with the corresponding endosomal masks and integrating the result for each cell. The initial integrated intensity of EphA-mCitrine loading profiles was normalized for each cell.

For quantifying the colocalization between EphA2-mCitrine and dSH2-mCherry, ImageJ was used for intensity correlation analysis to derive Pearson's correlation coefficient (PCC). This is a numerical indicator of the strength of the linear relationship between two variables, *X* and *Y* as follows:


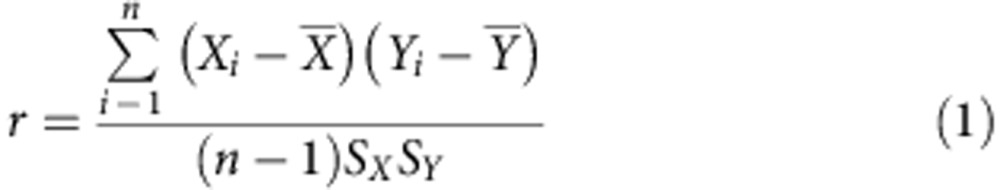


where (*r*) is PCC; *X*_*i*_ and *Y*_*i*_ are the fluorescence intensities (in arbitrary units) of EphA2-mCitrine and SH2-mCherry in pixel (*i*), (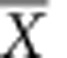) and (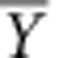) are the averages for the variable*s X* and *Y* and *S*_*X*_ and *S*_*Y*_ are the s.d.

### Fluorescence recovery after photobleaching and FLAP

Fluorescence recovery after photobleaching (FRAP) experiments were carried out at 37 °C on a Leica TCS SP5 DMI6000 confocal microscope (Leica Microsystems, Mannheim) equipped with a HCX PL APO × 63/1.4 NA oil objective (Leica Microsystems) and an environment-controlled chamber maintained at 37 °C. The FRAP image acquisition was divided into three steps: (1) pre-bleach, (2) bleaching and (3) post-bleach. In the pre-bleach step, a total of 10 fluorescence images for EphA2-mCitrine and bfp–Rab11a with a time interval of 10 sec were acquired, using the 514 nm Argon laser at 10% power (LGK 7872 ML05, Lasos) and the 405 nm Cube laser at 2% power (1162002/AF, Coherent), respectively. Bleaching of EphA2-mCitrine was performed with the 514 nm Argon laser at 100% power and bleaching was restricted to a region of interest (ROI) on the RE, identified by expression of bfp–Rab11a. In the post-bleach step, a total of 80 fluorescence images for EphA2-mCitrine and bfp–Rab11a with a time interval of 10 sec were acquired, using the 514 nm Argon laser at 10% power and the 405 nm Cube laser at 2% power, respectively. Images were background corrected in ImageJ and the relative fluorescence intensity *I*(*t*) was computed as follows:


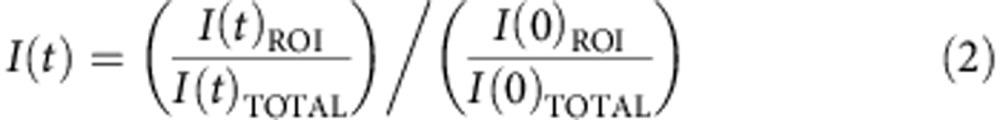


where *I*(*t*)_ROI_ is the average fluorescence intensity in the ROI at time *t*, *I*(*t*)_TOTAL_ is the average fluorescence intensity of the whole cell at the same time point, *I*(0)_ROI_ is the average fluorescence intensity in the ROI of the pre-bleach images, *I*(0)_TOTAL_ is the average fluorescence intensity of the whole cell of the pre-bleach images. The relative intensity was then fitted by a single exponential function:


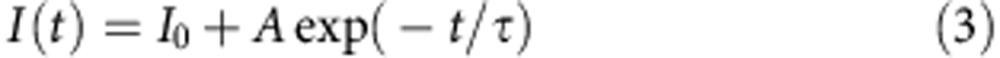



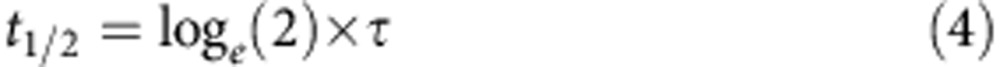


where *I*(*t*) is the fluorescence intensity (in arbitrary units) at time *t* (s), *I*_0_ is the residual intensity after photobleaching in the ROI, *τ* (s) is the exponential recovery time constant, *A* is the exponential amplitude, *t*_1/2_ (s) is the half time of fluorescence recovery.

FLAP experiments were carried out at 37 °C on a Leica TCS SP5 DMI6000 confocal microscope (Leica Microsystems, Mannheim) equipped with a HCX PL APO × 63/1.4 NA oil objective (Leica Microsystems). The FLAP image acquisition was divided into three steps: (1) pre-activation, (2) photoactivation and (3) post-activation. In the pre-activation step, a total of 3 fluorescence images for EphA2–paGFP and EphA2-mCherry with a time interval of 10 sec were acquired, using the 488 nm Argon laser at 10% power and the 561 nm DPSS laser at 11% power (YLK 6120 T02, Lasos), respectively. Photoactivation of EphA2–paGFP was performed with the 405 nm Cube laser at 80% power and photoactivation was restricted to a ROI on the RE, identified by co-expression of EphA2-mCherry. In the post-activation step, a total of 30 fluorescence images for EphA2–paGFP and EphA2-mCherry with a time interval of 40 sec were acquired, using the 488 nm Argon laser at 10% power and the 561 nm DPSS laser at 11% power, respectively. At the end of the experiment an image of bfp–Rab11a was acquired with 5% 405-laser power. Following background correction, FLAP at the RE was quantified as the ratio of local EphA2–paGFP to EphA2-mCherry fluorescence, to account for changes in the structure and intensity in the ROI. Fluorescence gain on the PM was quantified as the ratio of local (cell periphery) EphA2–paGFP to EphA2-mCherry fluorescence. For measuring half time of PM gain or RE loss of EphA2 fluorescence in the selected ROIs, normalized fluorescence decay cures were averaged and fitted by a single exponential function (Eqs. 3, 4).

### Anisotropy microscopy

Anisotropy microscopy was done at RT in imaging medium as described before^39^ in Cos-7 cells ectopically expressing donor-only LIFEA2 or EphA2-mCitrine. Images were acquired 15–24 h post-transfection, using an Olympus IX81 inverted microscope (Olympus, Hamburg, Germany) equipped with a MT20 illumination system. A linear dichroic polarizer (Meadowlark optics, Frederick, CO) was placed in the illumination path of the microscope, and two identical polarizers were placed in an external filter wheel at orientations parallel and perpendicular to the polarization of the excitation light. Fluorescence images were collected via a × 20/0.7 NA air objective using an Orca CCD camera (Hamamatsu Photonics, Hamamatsu City, Japan). For each measurement two images were taken, one with the emission polarizer oriented parallel to the excitation polarizer (***I***_II_) and one with the emission polarizer oriented perpendicular to the excitation polarizer (***I***_⊥_). Steady-state anisotropy (***r***^*i*^) was calculated in each pixel *i* by:


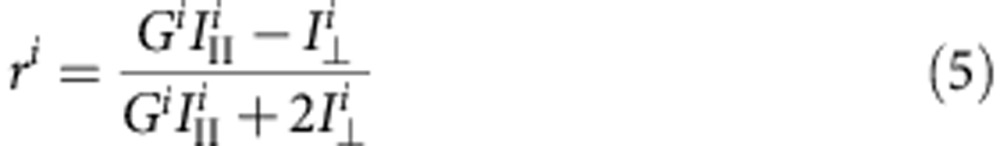


The G-factor (***G***^*i*^) was determined by acquiring the ratio of the intensities at perpendicular and parallel orientations for a fluorophore in solution (fluorescein) with a steady-state anisotropy close to zero. The CellR software supplied by the microscope manufacturer (Olympus, Hamburg, Germany) controlled data acquisition. Computed anisotropy images were displayed with false color-coding using ImageJ.

### FLIM

FLIM measurements were made on an Olympus FluoView FV1000 laser scanning confocal microscope equipped with a time-correlated single-photon counting module (LSM Upgrade Kit, Picoquant), using × 60/1.35 NA oil objective (Olympus). All pulsed lasers were controlled with the Sepia II software (PicoQuant GmbH) at pulse repetition frequency of 40 MHz. The sample was excited using a 470 nm diode laser (LDH 470, Picoquant). Fluorescence emission was spectrally filtered using a narrow-band emission filter (HQ 525/15, Chroma). Photons were detected using a single-photon counting avalanche photodiode (PDM Series, MPD) and timed using a single-photon counting module (PicoHarp 300, Picoquant). Using the SymPhoTime software V5.13 (Picoquant), images were collected after an integration time of ∼3 min with ∼3.0–5.0 × 10^6^ photons. Data were analysed with precision FLIM analysis code as described in (ref. 30). The analysis code uses a parameterized model to account for the instrument response function. In case of FRET, the measured biexponential decay profile in each pixel becomes the sum of the two decay profiles scaled by their respective populations. The normalized fluorescence decay profile *I*(*t*)/*I*(0) as function of time (*t*) was thus fitted to a double-exponential decay model:


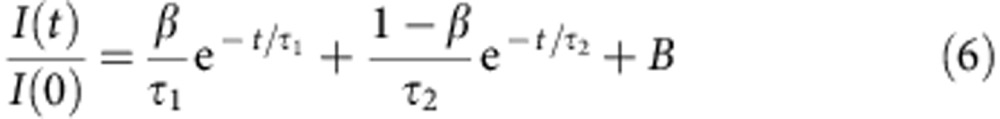


where *B* is background fluorescence, *β* is the fractional photon contribution and *τ*_*i*_ the fluorescence lifetime of component *i* (*i=*1,2), The average fluorescence lifetime *τ* was computed as follows:


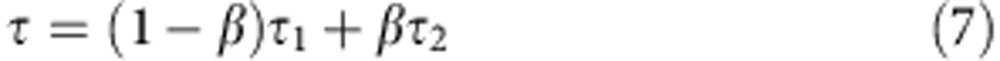


*τ*-maps obtained by pFLIM were displayed in pseudo-color using IgorPro.

### Statistical analysis

All results are expressed as mean±s.e.m. Statistical significance was estimated either by the *F*-test, Kolmogorov–Smirnov nonparametric *t*-test or Unpaired Student's *t*-test. Significance level is indicated as follows: *P*<0.05 (*), *P*<0.01 (**), *P*<0.001 (***) and *P*<0.0001 (****). The *P* value for the *F*-test was calculated as an average of the *P* values computed from the multiple comparisons between the regression lines, using a Monte Carlo approach.

## Additional information

**How to cite this article:** Sabet, O. *et al.* Ubiquitination switches EphA2 vesicular traffic from a continuous safeguard to a finite signalling mode. *Nat. Commun.* 6:8047 doi: 10.1038/ncomms9047 (2015).

## Supplementary Material

Supplementary Information Supplementary Figures 1-8, Supplementary Note 1, Supplementary Methods and Supplementary References

Supplementary Movie 1 Confocal time-lapse sequence showing the spatial distribution of EphA2 and Rab5 upon stimulation of Cos-7 cells with ephrinA1-Fc. Ectopically expressed EphA2-mCitrine (left column, inverted grey scale), mCherry-Rab5 (middle column, inverted grey scale) and an RGB overlay of EphA2-mCitrine (green), mCherry-Rab5 (magenta). Cos-7 cells were stimulated with pre-clustered ephrinA1-Fc, 2 μg ml-1 immediately after the first frame and imaged every 40 sec.

Supplementary Movie 2 Confocal time-lapse sequence showing the spatial distribution of Y813F-EphA2 and Rab5 upon stimulation of Cos-7 cells with ephrinA1-Fc. Ectopically expressed Y813F-EphA2-mCitrine (left column, inverted grey scale), mCherry-Rab5 (middle column, inverted grey scale) and an RGB overlay of EphA2-mCitrine (green), mCherry-Rab5 (magenta). Cos-7 cells were stimulated with pre-clustered ephrinA1-Fc, 2 μg ml-1 immediately after the first frame and imaged every 40 sec.

Supplementary Movie 3 Confocal time-lapse sequence showing the spatial distribution of EphA2 and Rab5 upon treatment with 0.33 mM pervanadate. Ectopically expressed EphA2-mCitrine (left column, inverted grey scale), mCherry-Rab5 (middle column, inverted grey scale) and an RGB overlay of EphA2-mCitrine (green), mCherry-Rab5 (magenta). Cos-7 cells were treated with a freshly prepared pervanadate solution (0.33 mM) immediately after the first frame and imaged every 40 sec.
