# Supplementary material for: Ubiquitination switches EphA2 vesicular traffic from a continuous safeguard to a finite signalling mode
Source: Nat Commun. 2015 Aug 21;6:8047. doi: 10.1038/ncomms9047 (PMC4560775; doi:10.1038/ncomms9047)
Supplement: Supplementary Information — Supplementary Figures 1-8, Supplementary Note 1, Supplementary Methods and Supplementary References [file ncomms9047-s1.pdf]

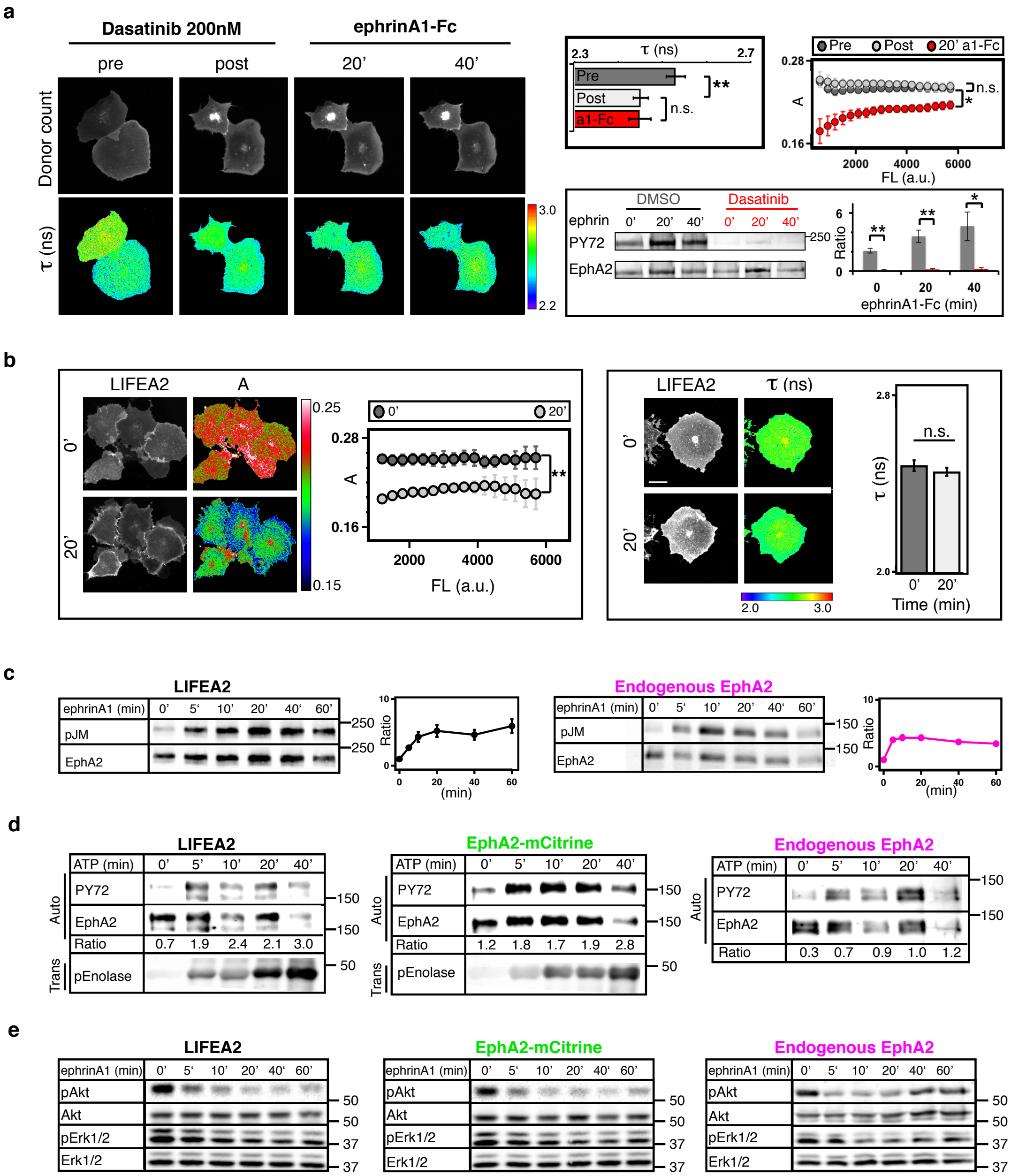

Supplementary Figure 1: Functional characterization of LIFEA2. (a) Ligand-clustering does not induce intermolecular FRET when dasatinib locks LIFEA2 in an active conformation. Left images: LIFEA2 photon count (donor count), τ (ns; color-coding right), pre-, post-dasatinib (200 nM, 1 h) and post-addition of pre-clustered ephrinA1-Fc (2 μg ml^{-1}, time in min. Right graphs: upper-left: average τ ± s.e.m. (n= 3 independent experiments, 9 cells) [**, P<0.01; n.s. not significant; unpaired *t*-test],

upper-right: 2D-histogram of anisotropy (A*)* ± s.e.m. versus fluorescence (FL) of LIFEA2 [*, P<0.05; n.s. not significant; *F*-test; n= 3 independent experiments]. Legend: pre- (dark grey), post-dasatinib (light grey), 20 min clustered ephrinA1-Fc (red). Lower panel: anti-GFP immunoprecipitated (IP) LIFEA2 probed for phosphotyrosine (PY72) and EphA2 in the presence of DMSO (grey) or 200 nM dasatinib (red). Ratio of PY72/EphA2 ± s.e.m. [n=3 blots, *, P<0.05; **, P<0.01; unpaired *t*-test]. **(b)** Dimerization of LIFEA2 with unclustered ephrinA1-Fc does not induce intermolecular FRET. Left-box: LIFEA2 fluorescence and anisotropy (A*,* color-coding right) of donor-only LIFEA2 pre- and 20 min post-unclustered ephrinA1-Fc (500 ng ml^{-1}). Graph: corresponding 2D-histogram of A (mean ± s.e.m.) versus FL [n= 30 cells, **, P<0.01; *F*-test;]. Right-box: LIFEA2 fluorescence and τ (ns; color-coding below) pre- and post-unclustered ephrinA1-Fc (500 ng/ml, time: min). Plot: LIFEA2 τ ± s.e.m. (3 independent experiments, n=12 cells) (n.s. not significant; unpaired *t*-test) **(c)** JMS phosphorylation of LIFEA2 and endogenous EphA2 in Cos-7 cells upon ephrinA1-Fc (2 μg ml^{-1}) addition (time: min). In both cases, IPs were probed for phospho-JMS-tyrosine (pJM) and EphA2. Graphs: ratio of pJM/EphA2 ± s.e.m. (n= 3 blots). **(d)** *In vitro* kinase assay of LIFEA2 (IP: anti-GFP), EphA2-mCitrine (IP: anti-GFP) and endogenous EphA2 in MDA-MD-231 cells (IP: anti-EphA2) upon addition of 1mM ATP (time: min). Blots probed for PY72 and EphA2 to show autophosphorylation ratio (PY72/EphA2) and transphosphorylation of enolase (pEnolase). **(e)** Downstream signalling of LIFEA2, EphA2-mCitrine and endogenous EphA2 in Cos-7 cells after ephrinA1-Fc (2 μg ml^{-1}) addition for the indicated time (min). Blots were probed for phospho-Akt (S473) (pAkt), total Akt (Akt), phospho-Erk1/2 (Thr 202/ Tyr 204) (pErk 1/2) and total Erk1/2 (Erk 1/2).

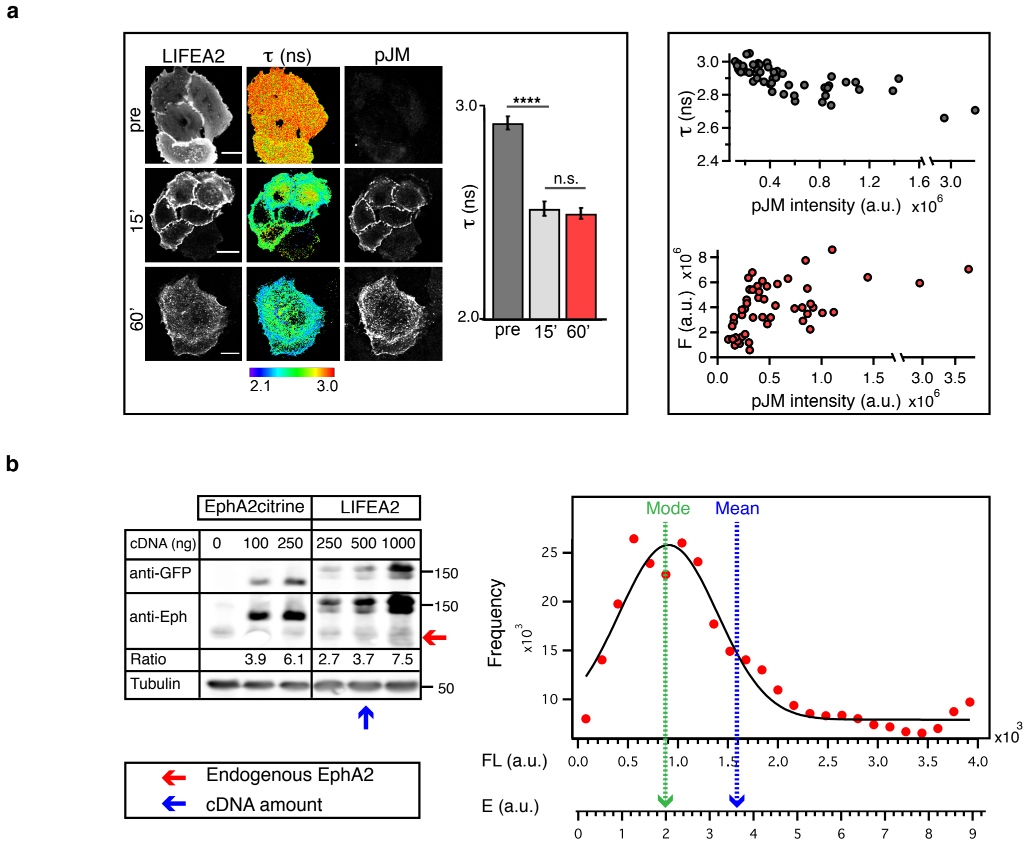

Supplementary Figure 2: Phosphorylation of LIFEA2. (a) The change in fluorescence lifetime (τ) of LIFEA2 correlates with JMS phosphorylation. Left box: Cos-7 cells expressing LIFEA2 were stained for pJM following stimulation with pre-clustered ephrinA1-Fc (2 μg ml^{-1}) at the indicated time (min). 1st column: mCitrine fluorescence images, 2nd: τ (ns; color-coding below), 3rd: fluorescence images of pJM. Bar graph: LIFEA2 τ (ns) ± s.e.m. in cell areas positive for pJM [****, P<0.0001; n.s. not significant; unpaired *t*-test]. Right box: 2D histograms: upper; LIFEA2 τ versus pJM integrated fluorescence intensity (pJM intensity), lower; LIFEA2 integrated fluorescence intensity (F) versus pJM intensity. Note: mCitrine fluorescence lifetime at 25 °C (fixed cells) is higher than at 37 °C [1]. Data obtained from n=51 cells. Scale bars: 10 μm. **(b)** Quantification of LIFEA2 expression level. Left: blots of EphA2-mCitrine, LIFEA2 and endogenous EphA2 in Cos-7 cells probed with anti-GFP, anti-Eph and anti-tubulin. The

ratio of expressed EphA2/endogenous EphA2 is depicted below each condition. Red arrow denotes the band of endogenous EphA2 and blue arrow denotes the amount of LIFEA2 cDNA used in dose-response experiments depicted in figure 2a. Right: fluorescence intensity distribution (FID) of LIFEA2 accumulated from all pixels in many cells. Upper x-axis, fluorescence intensity (FL; a.u.) and lower x-axis, relative expression level. Mean fluorescence from the FID and average expression level as determined by western blot (blue). The mode of the asymmetric FID and the corresponding expression level (green).

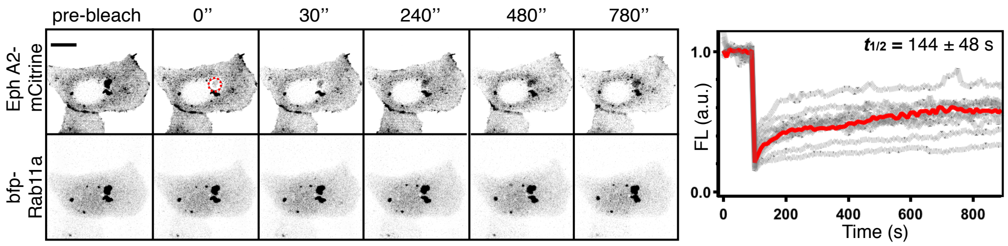

Supplementary Figure 3: Trafficking of EphA2 to the recycling endosome. Fluorescence recovery after selective photobleaching of EphA2-mCitrine on the recycling endosome (RE) in Cos-7 cells. Upper row: EphA2-mCitrine fluorescence at the indicated time (s) after photobleaching on the RE (red circle), lower row: bfp-Rab11a fluorescence. Scale bars: 10 µm. Right graph: relative fluorescence increase of EphA2-mCitrine (FL) at the RE. Pre-bleach fluorescence was normalized to one. Recovery curves (10 experiments, grey) were fitted individually to an exponential function to retrieve the half-time of fluorescence recovery on the RE ($t_{1/2}$ = 144 ± 48 s). Recovery curve of representative images is depicted in red.

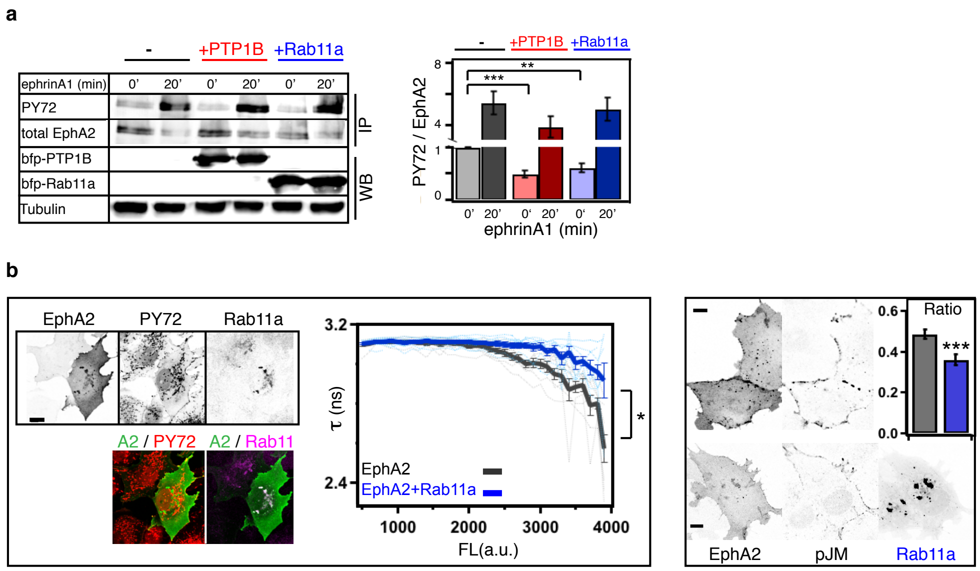

Supplementary Figure 4: Vesicular recycling supresses autonomous EphA2 activity. **(a)** Ectopic expression of Rab11a or PTP1B suppresses basal phosphorylation of endogenous EphA2. Left: western blot of anti-Eph IP without (-) or in the presence of ectopically expressed bfp-PTP1B (+PTP1B) or bfp-Rab11a (+Rab11a). Blots were probed with anti-phospho-tyrosine (PY72), anti-EphA2 (EphA2), anti-bfp (bfp-PTP1B, bfp-Rab11a) and anti-tubulin (Tubulin) as loading control. Right graph: corresponding quantification of mean phosphorylation (PY72/EphA2) ± s.e.m. from n=6 blots [***, P<0.001, **, P<0.01; unpaired *t*-test]. **(b)** Phosphorylated fraction of EphA2-mCitrine is lowered by ectopic expression of bfp-Rab11a. Left box: left, fluorescence images of EphA2-mCitrine (EphA2, 1st), Cy3.5 labelled anti-phospho-tyrosine antibody (PY72, 2nd) and bfp-Rab11a (Rab11a, 3rd) in fixed Cos-7 cells; lower row: overlay; right graph, quantification of intermolecular FRET by FLIM between EphA2-mCitrine and Cy3.5 labelled PY72 in absence (black) or presence (blue) of bfp-Rab11a; EphA2-mCitrine τ ± s.e.m. (ns) versus its fluorescence intensity (FL) from n=38 cells [*, P<0.05; non-

parametric Wilcoxon-rank test]. The increase in τ as function of FL indicates less phosphorylation of EphA2-mCitrine upon ectopic expression of Rab11a. Right box: Phosphospecific staining of EphA2-JMS shows less phosphorylation of JMS tyrosines upon ectopic expression of bfp-Rab11a. Fluorescence images of EphA2-mCitrine (EphA2, first column:), immunostained phosphorylated JMS-tyrosines (pJM, middle column) and bfp-Rab11a (Rab11a, last column). Inset: corresponding ratiometric quantification of phosphorylated EphA2-mCitrine on the periphery of the cell over total amount of phosphorylated EphA2-mCitrine in absence (black) or presence (blue) of bfp-Rab11a [mean ± s.e.m.; *** (p= 0.001) unpaired t-test, n= 20 cells]. Scale bars: 10 µm.

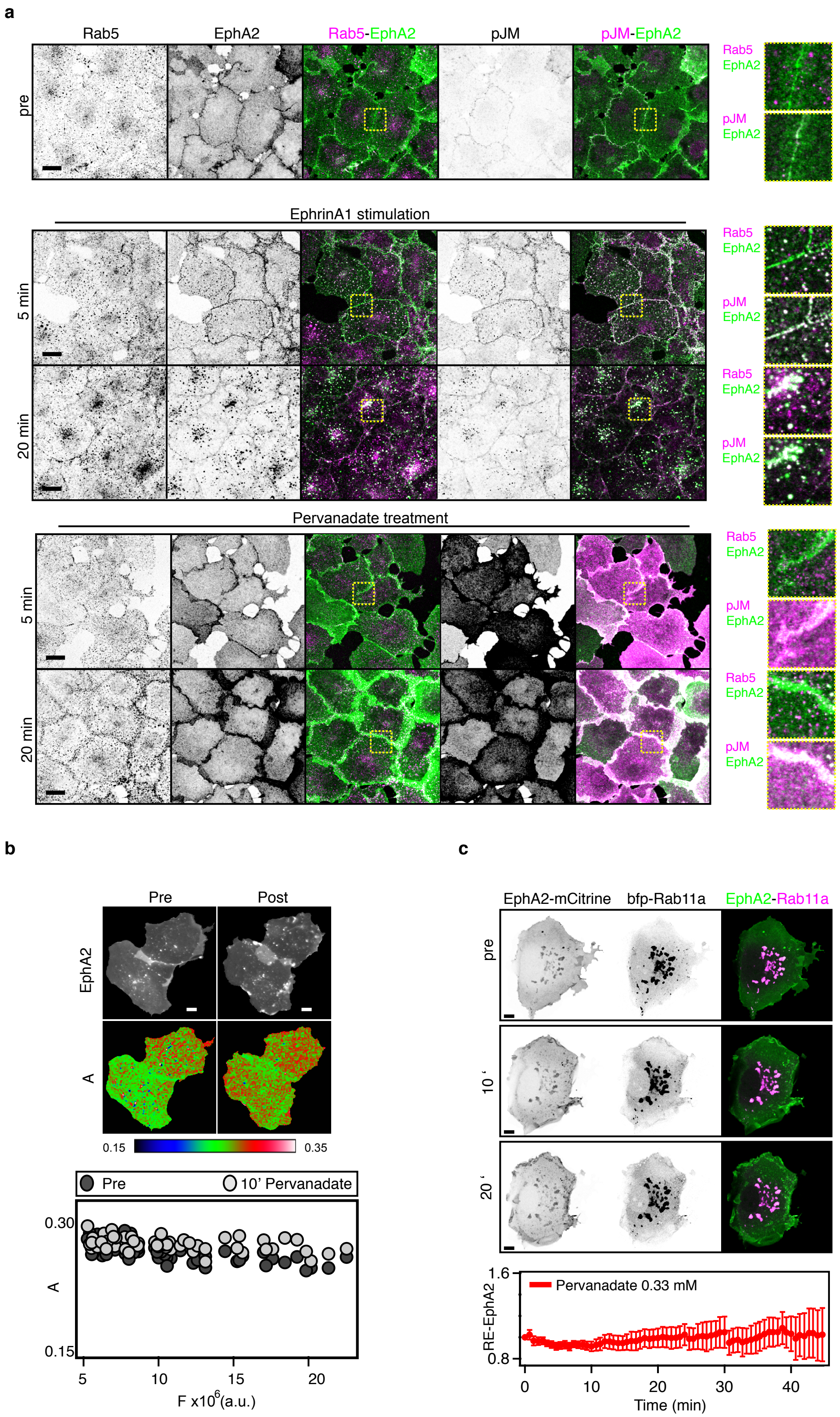
a
Rab5
EphA2
Rab5-EphA2
pJM
pJM-EphA2
pre
EphrinA1 stimulation
5 min
20 min
Pervanadate treatment
b
Pre
Post
EphA2
A
0.15
0.35
Pre
10' Pervanadate
0.30
0.15
5
10
15
20
F x10^6 (a.u.)
c
EphA2-mCitrine
bfp-Rab11a
EphA2-Rab11a
10 '
20 '
Pervanadate 0.33 mM
1.6
0.8
RE-EphA2
0
10
20
30
40
Time (min)

Supplementary Figure 5: Differential vesicular trafficking of ligand- versus autonomously-activated EphA2. (a) Co-localization of endogenous EphA2, Rab5 and phosphorylated JMS (pJM) in fixed Cos-7 cells**.** Immunostaining of Rab5 (1st column), EphA2 (2nd column), EphA2 (green) and Rab5 (magenta) overlay (3rd column), pJM (4th column), EphA2 (green) and pJM (magenta) overlay (5th column). Upper panel, pre-stimulation, middle: 5 min (1st row) and 20 min (2nd row) post stimulation with preclustered ephrinA1-Fc, 2 μg ml^{-1}, lower: 5 min (1st row) and 20 min (2nd row) post treatment with 0.33 mM pervanadate. Right-side panels represent a magnified image from respective overlay ROIs (yellow boxes). **(b)** EphA2 activated by pervanadate remains unclustered. Upper images: representative fluorescence- (1st row) and anisotropy-images (2nd row, (A), color-coding below) of EphA2-mCitrine expressed in Cos-7 cells. First column: pre-, second: 10 min post-treatment with 0.33 mM pervanadate. Lower graph: 2D-histogram of A versus integrated fluorescence (F*)* for individual cells (n=62 cells) pre- (black circles) and 10 min post-pervanadate (grey circles). **(c)** Time-lapse images of EphA2 and Rab11a co-localization in Cos-7 cells upon treatment with 0.33 mM pervanadate. Upper panels: fluorescence images of EphA2-mCitrine (left-column), bfp-Rab11a (middle), EphA2-mCitrine (green) and bfp-Rab11a (magenta) overlay (last column). Lower graph: ratiometric analysis of EphA2-mCitrine on the Rab11-positive RE after pervanadate treatment (mean ± s.e.m., n=6 cells). Scale bars: 10 μm.

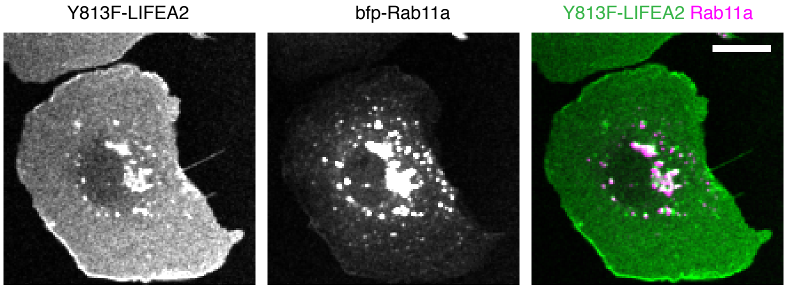

Supplementary Figure 6: Co-localization of Y813F-LIFEA2 and Rab11a. Fluorescence images of ectopically expressed Y813F-LIFEA2 (first) and bfp-Rab11a (second) in living Cos-7 cells, green/magenta overlay (third). Scale bars: 10 µm.

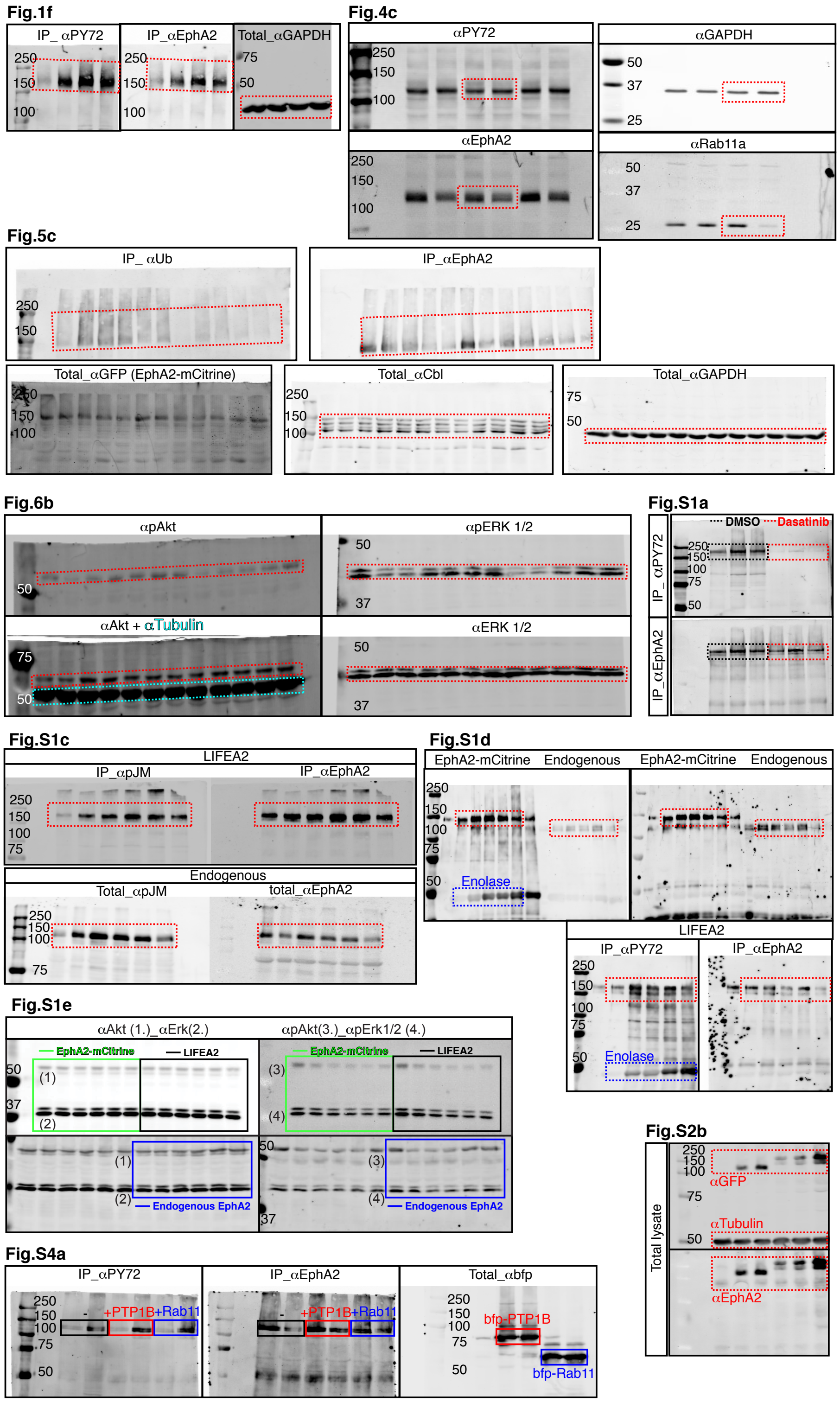

Supplementary Figure 7: Unedited scans of western blots. Cropped regions are indicated with a rectangle as appropriate. Antibodies are provided for each blot.

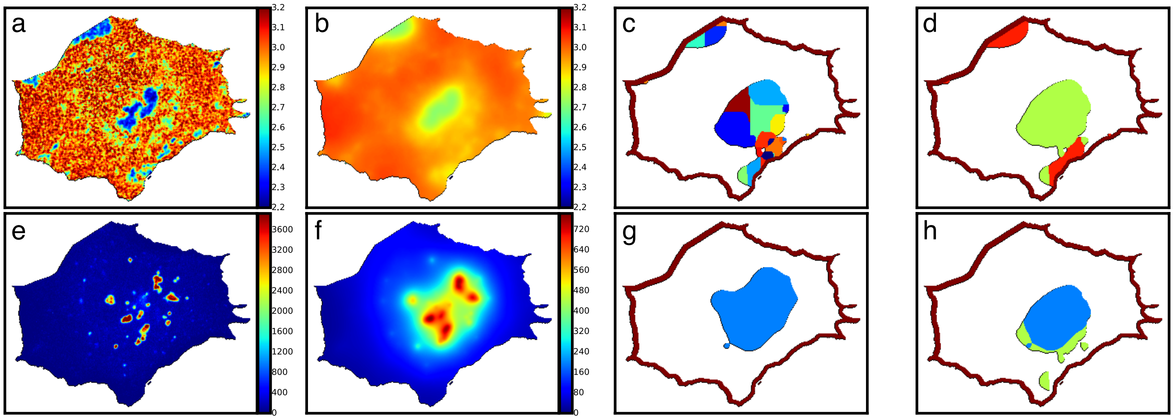

Supplementary Figure 8: Analysis steps undertaken to calculate the percentage of overlap between cytosolic areas with EphA2/PTP1B-D/A interactions and the RE. Color bar denotes EphA2-mCitrine fluorescence lifetime (upper row) or bfp-Rab11a intensity (lower row). See also Supplementary Methods.

Supplementary Note 1

We ascertained by three independent experimental approaches that the change in the fluorescence lifetime of LIFEA2 reports on a change in receptor conformation (intramolecular FRET) and not clustering (intermolecular FRET). The first approach is detailed in the main article (Fig.1d).

Small-molecule inhibitors of RTKs lock the kinase domain (KD) in a specific conformation, be it the active conformation (e.g. Dasatinib) [2,3], or the inactive one (e.g. Imatinib) [4,5]. We therefore used 200 nM of the ATP competitive inhibitor Dasatinib [6] to lock the KD of LIFEA2 expressed in Cos7 cells into an active conformation. This resulted in an expected shift of the fluorescence lifetime (τ) of LIFEA2 towards lower values. Further addition of clustered ephrinA1-Fc induced receptor clustering as seen by a drop in the fluorescence anisotropy of mCitrine but did not change the fluorescence lifetime of LIFEA2 locked in a dasatanib-induced conformation (Supplementary Fig, 1a). The dasatanib induced change in fluorescence lifetime shows that LIFEA2 reports on conformational changes in the receptor, whereas the ephrinA1-Fc induced clustering of the dasatanib-locked receptor confirms that the fluorescence lifetime of LIFEA2 is not sensitive to clustering.

Unclustered, dimeric Fc-ephrinA1 was used to disentangle dimerization- from conformation-coupled changes in the fluorescence lifetime of LIFEA2. Contrary to other RTKs, dimerization of EphA2 by ephrinA1-Fc is not sufficient for receptor activation [7,8]. In Cos-7 cells expressing LIFEA2 without mCherry (donor-only LIFEA2), unclustered ephrinA1-Fc increased the extent of homo-FRET between mCitrine as measured by fluorescence anisotropy [9,10] (Supplementary Fig. 1b). This molecular proximity of the

mCitrine in the JMS is indicative of receptor dimerization. However, in FLIM experiments, unclustered ephrinA1-Fc failed to change the fluorescence lifetime of LIFEA2, showing that intermolecular FRET between mCitrine and mCherry was not taking place in LIFEA2 dimers (Supplementary Fig. 1c).

Supplementary Methods

Calculating the percentage of overlap between cytosolic areas with high EphA2 -mCitrine/ m-CherryPTP1B-D181A interaction and the recycling endosomes

Two distinct sites of significant EphA2/PTP1B-D181A interaction were observed: stabilized cell-cell contact points and the perinuclear region. Here, we estimated the spatial coincidence between the perinuclear area with high EphA2/PTP1B-D181A interaction and the areas in the vicinity of Rab11-positive recycling endosomes (REs). To quantify the coincidence per cell, EphA2-mCtirine fluorescence lifetime (Supplementary Fig.8a) and bfp-Rab11a fluorescence images (Supplementary Fig.8e) were analyzed as follows:

1. Smoothening of EphA2-mCitrine fluorescence lifetime (Supplementary Fig.8b) and bfp-Rab11a fluorescence intensity images (Supplementary Fig.8f) to obtain an estimation of their densities by a consensus-like algorithm (detailed below).
2. Using their respective density estimations, areas with significantly low EphA2-mCitrine lifetime (Supplementary Fig.8c) and high bfp-Rab11a fluorescence intensity (Supplementary Fig.8g) were identified using Otsu's method for thresholding [11]. Areas with high bfp-Rab11a intensity designate vicinity to the REs, and are used in the final step.

3. As the density estimation is a fairly smooth function, its local minima pixels (pixels with lower density values than those of all neighboring pixels) were clearly detected. To distinguish between perinuclear areas and cell-cell contact points that are in close proximity, the basins of attraction around each locally minimal pixel were identified (Supplementary Fig.8c). Pixel *j* is in the basin of attraction of a locally minimal pixel *i*, if one can move from *j* to *i* following the steepest descent along the density gradient.
4. The basins that border with the edges of the cell and thereby define cell-cell contact points were removed (Supplementary Fig.8d, red areas).
5. This leaves the desired cytosolic (perinuclear) areas with high EphA2/PTP1B-D181A interactions (Supplementary Fig.8d, green areas). However, in cases where only a small number of nonspecific pixels remained in the end, those cells were removed.
6. The overlap between the high bfp-Rab11a intensity areas and the perinuclear areas with low EphA2-mCitrine lifetime was represented as a percentage from the total area with low EphA2-mCitrine lifetime (Supplementary Fig.8h).

The percentage of interacting EphA2/PTP1B in the perinuclear area that coincided with areas of Rab11-rich endosomes was calculated from 59 cells (81±17%, mean ± std.). This was compared to the expected overlap percentage, calculated as a ratio of the size of the high Rab11-intensity area to the total size of the cell (25±13%).

Density estimation by a consensus-like algorithm: Given a snapshot image of a cell, from its pixel values we can observe how a quantity of interest (EphA2-mCitrine lifetime or

bfp-Rab11a intensity) is spatially arranged. Due to fluctuations in the cell in each snapshot a non-smooth spatial arrangement is observed. Nevertheless, the images reveal some patterns, thus it can be presumed that each snapshot is a random realization of an underlying smooth density function. Estimating this function is of interest, as it can be easily compared to density functions of other quantities from the same cell.

For each pixel i, we denote with N_i the set of its neighboring pixels from the cell and with $|N_i|$ the size of N_i, i.e. the number of neighboring pixels of i. Typically $|N_i|$ is 4, except for the pixels that lie on the edges of the cell. Each pixel i has an initial value $x_i(0)$, which depicts the value from the snapshot image. In order to make smoother density estimation, we aim to accommodate the differences between the values of neighboring pixels. Therefore, we assume that each pixel has a cost function of the form

$$C(x_i, x_{N_i}) = \frac{1}{2}\sum_{j \in N_i}(x_i - x_j)^2 + \frac{1}{2}K(x_i - x_i(0))^2 \tag{1}$$

that it tries to minimize. $K \geq 0$ is a parameter that represents the "stubbornness" of every pixel regarding its initial value. Higher values of K lead to larger stubbornness, hence the final density estimate will be similar to the initial arrangement, thus non-smooth. For $K = 0$ consensus will be reached among the pixels, and all of them will have the same value in the final estimate. In our analysis we use $\mathrm{K} = 0.005$ to obtain a fairly smooth density estimate. An iterative consensus-like algorithm is applied for minimization of the cost function, similar to the one described in [12]. For every pixel i the estimate of the value for the next time step is

$$x_i(t+1) = \frac{1}{|N_i| + K}\sum_{j \in N_i} x_j(t) + \frac{K}{|N_i| + K} x_i(0) \tag{2}$$

This algorithm converges exponentially fast to our desired density estimate.

Supplementary References

1 Walther, K. A., Papke, B., Sinn, M. B., Michel, K. & Kinkhabwala, A. Precise measurement of protein interacting fractions with fluorescence lifetime imaging microscopy. *Mol. Biosyst.* **7**, 322-336 (2011).

2 Tokarski, J. S. *et al*. The structure of Dasatinib (BMS-354825) bound to activated ABL kinase domain elucidates its inhibitory activity against imatinib-resistant ABL mutants. *Cancer Res.* **66**, 5790-5797 (2006).

3 Farenc, C., Celie, P. H. N., Tensen, C. P., de Esch, I. J. P. & Siegal, G. Crystal structure of the EphA4 protein tyrosine kinase domain in the apo- and dasatinib-bound state. *FEBS Letters* **585** (2011).

4 Mol, C. D. *et al*. Structural basis for the autoinhibition and STI-571 inhibition of c-Kit tyrosine kinase. *J Biol Chem* **279**, 31655-31663 (2004).

5 Nagar, B. *et al*. Crystal structures of the kinase domain of c-Abl in complex with the small molecule inhibitors PD173955 and imatinib (STI-571). *Cancer Res.* **62**, 4236-4243 (2002).

6 Chang, Q., Jorgensen, C., Pawson, T. & Hedley, D. W. Effects of dasatinib on EphA2 receptor tyrosine kinase activity and downstream signalling in pancreatic cancer. *Br. J. Cancer* **99**, 1074-1082 (2008).

7 Lawrenson, I. D. *et al*. Ephrin-A5 induces rounding, blebbing and de-adhesion of EphA3-expressing 293T and melanoma cells by CrkII and Rho-mediated signalling. *J. Cell Sci.* **115** (2002).

8 Davis, S. *et al*. Ligands for EPH-related receptor tyrosine kinases that require membrane attachment or clustering for activity. *Science* **266**, 816-819 (1994).

9 Varma, R. & Mayor, S. GPI-anchored proteins are organized in submicron domains at the cell surface. *Nature* **394** (1998).

10 Bader, A. N. *et al*. Homo-FRET imaging as a tool to quantify protein and lipid clustering. *Chemphyschem* **12**, 475-483 (2011).

11 Otsu, N. A threshold selection method from gray-level histograms. *Automatica* **11**, 23-27 (1975).

12 Ghaderi, J. & Srikant, R. in *American Control Conference (ACC), 2013*. 1982-1987 (IEEE).
